# Topographic correlates of driver mutations and endogenous gene expression in pediatric diffuse midline gliomas and hemispheric high-grade gliomas

**DOI:** 10.1038/s41598-021-92943-0

**Published:** 2021-07-13

**Authors:** Eve Kazarian, Asher Marks, Jin Cui, Armine Darbinyan, Elizabeth Tong, Sabine Mueller, Soonmee Cha, Mariam S. Aboian

**Affiliations:** 1 Department of Radiology & Biomedical Imaging, Yale School of Medicine, New Haven, CT USA; 2 Department of Pediatric Hematology & Oncology, Yale School of Medicine, New Haven, CT USA; 3 Department of Neuropathology, Yale School of Medicine, New Haven, CT USA; 4 Department of Radiology, , University of California, San Francisco, San Francisco, USA; 5 Division of Pediatric Hematology & Oncology, Department of Pediatrics, University of California, San Francisco, San Francisco, USA; 6 Department of Neurological Surgery, University of California, San Francisco, San Francisco, USA; 7 Department of Neurology, University of California, San Francisco, San Francisco, USA

**Keywords:** Genomic analysis, Imaging

## Abstract

We evaluate the topographic distribution of diffuse midline gliomas and hemispheric high-grade gliomas in children with respect to their normal gene expression patterns and pathologic driver mutation patterns. We identified 19 pediatric patients with diffuse midline or high-grade glioma with preoperative MRI from tumor board review. 7 of these had 500 gene panel mutation testing, 11 patients had 50 gene panel mutation testing and one 343 gene panel testing from a separate institution were included as validation set. Tumor imaging features and gene expression patterns were analyzed using Allen Brain Atlas. Twelve patients had diffuse midline gliomas and seven had hemispheric high-grade gliomas. Three diffuse midline gliomas had the K27M mutation in the tail of histone H3 protein. All patients undergoing 500 gene panel testing had additional mutations, the most common being in ACVR1, PPM1D, and p53. Hemispheric high-grade gliomas had either TP53 or IDH1 mutation and diffuse midline gliomas had H3 K27M-mutation. Gene expression analysis in normal brains demonstrated that genes mutated in diffuse midline gliomas had higher expression along midline structures as compared to the cerebral hemispheres. Our study suggests that topographic location of pediatric diffuse midline gliomas and hemispheric high-grade gliomas correlates with driver mutations of tumor to the endogenous gene expression in that location. This correlation suggests that cellular state that is required for increased gene expression predisposes that location to mutations and defines the driver mutations within tumors that arise from that region.

## Introduction

Pediatric high-grade gliomas are aggressive tumors with different molecular characteristics than adult gliomas^1–3^. There is a distinct difference between the driver mutations found in pediatric diffuse midline gliomas and hemispheric high-grade gliomas. Diffuse midline gliomas represent 10–15% of all childhood brain tumors and have median survival of 9–12 months^4^. MR imaging plays a critical role in diagnosis and surgical biopsy may not always be performed or feasible for diffuse midline gliomas in eloquent location. Radiation therapy is the standard of care due to their location within critical structures along the midline of the brain, yet chemotherapy and convection enhanced delivery approaches have been shown to play a role in treatment^5,6^. Many of these tumors contain somatic mutations in genes encoding histone tails; in 2016, the World Health Organization introduced the new classification of the diffuse midline gliomas, H3 K27M-mutant^7^. Diffuse midline gliomas, H3 K27M- mutant, frequently appear along with mutations in signal transduction (*ACVR1*), chromatin remodeling (*ATRX*), and cell cycle regulation (*TP53, PPM1D*)^4^. Recent genomic analyses have led to the development of subtype-specific therapies^6,8^. On the other hand, hemispheric high-grade gliomas in children commonly have hypermutated molecular signatures including *TP53, NF1, SETD2, ATRX*, *PTEN, ARID1A*, and *POLE*^4,6,9^. The survival rates for these tumors are poor, with 5-year survival rates of less than 20%. Maximum safe resection and radiation therapy in combination contribute to improved survival in patients with hemispheric high-grade gliomas as compared to those with diffuse midline gliomas^10,11^.

Pediatric high-grade gliomas can be distinguished by their location of origin; hemispheric high-grade gliomas originate in the cerebral hemispheres and diffuse midline gliomas originate in the midline structures, commonly in the pons, thalamus, or spinal cord^8,10,12–15^. In this study, we characterized the genetic mutations in 19 pediatric high-grade gliomas and correlated the location of tumors to endogenous gene expression within the region of tumor origin. Knowing the exact locations of the tumors and their genetic mutations allowed us to form a hypothesis that normal gene expression signature within particular part of the brain can define the driver mutations of tumors that arise from that anatomic location. A driver mutation is an alteration that gives a cancer cell a fundamental growth advantage for its neoplastic transformation. It differs from passenger mutations in that these do not necessarily determine the development of the cancer^16^. Previous studies have shown that chromatin structure affects distribution and types of mutations in cancer^16^. To test this hypothesis, we analyzed normal gene expression profile within different parts of the brain using microarray data from publicly available Allen Brain Atlas and correlated expression patterns of genes that were characterized to be driver mutations in high-grade gliomas that originated from these locations. The purpose of our study was to characterize the endogenous gene expression profile in anatomic locations where pediatric high-grade gliomas occur and correlate the gene expression level to susceptibility for developing a mutation, i.e., presence of tumors within this location with driver mutations within this gene. The Allen Brain Atlas provides data for normal intracranial gene expression in human adult and developmental brain (www.brain-map.org). This correlation would suggest that presence of chromatin microenvironment that is required for increased gene expression predisposes that location to mutations and defines the driver mutations within a specific region of the brain that led to tumor formation. Confirmation of gene expression patterns within specific regions of the brain associated with the presence of high-grade gliomas will lead to a better understanding of key genetic drivers involved in brain tumorigenesis.

## Methods

### Patient characteristics

A total of nineteen patients were either originally treated or seen for a second opinion regarding treatment options at two tertiary hospitals^6^. Informed consent was obtained prior to genetic sequencing for use of patient samples. Informed consent was obtained for all the subjects who are under 18 from a parent and/or legal guardian. This study was reviewed and approved by the Yale University and University of San Francisco institutional internal review boards. All methods were carried out in accordance with relevant guidelines and regulations.

### Characterization of tumor molecular profile

Genomic DNA was extracted from peripheral blood and tumor tissue micro-dissected from formalin-fixed, paraffin-embedded blocks. Capture-based next-generation sequencing (NGS) was performed at the Clinical Cancer Genomics Laboratory within our institution, using an assay targeting the coding regions of 510 cancer-related genes, *TERT* promoter, select introns from 40 genes (for detection of gene fusions and other structural variants), and intergenic regions at regular intervals along each chromosome (for chromosomal copy number assessment), altogether with a total sequencing footprint of 2.8 Mb^6^.

Sequencing libraries were prepared from genomic DNA with target enrichment performed by hybrid capture using a custom oligonucleotide library. Sequencing of the 500 gene panel assay was performed on an Illumina HiSeq 2500. Duplicate sequencing reads were removed computationally to allow for accurate allele frequency determination and copy number estimates. The analysis was based on the human reference sequence build hg19 (NCBI build 37), using the following software packages: BWA, Samtools, Picard tools, GATK, CNVkit, Pindel, SATK, Annovar, Freebayes, Delly, and Nexus Copy Number. Single nucleotide variants and small insertions/deletions (indels) were visualized and verified using Integrated Genome Viewer^6^. For samples with at least 25% tumor, where > 200 × coverage for the tumor sample and > 100 × coverage for the normal sample, the fully clonal single nucleotide variant sensitivity is 99% and small indel sensitivity is 83% while the specificity is 98% and 71%, respectively. Large insertions, deletions, and gene rearrangements may be detected but have only been individually validated for select examples. 50 gene panel testing was performed at Tumor Profiling Laboratory of Yale-New Haven Hospital under regulations of Clinical Laboratory Improvement Amendments of 1988 (CLIA). 343 gene panel testing was performed by Foundation One (Foundation Medicine Inc).

Molecular pathologists with specialization in neuropathology and brain tumor genetics organized results into formal reports, which detailed somatic and germline alterations as well as association with any known tumor predisposition syndromes, diagnostic or prognostic implications, and potential targeted therapies. Results were discussed at weekly multidisciplinary molecular tumor boards that included surgical and molecular pathologists together with oncologists, surgeons, and radiation oncologists from a wide variety of specialties^6^.

### Gene expression profile analysis

Gene expression profiles for *PPM1D*, *ASXL1*, *BCORL1*, *ACVR1, PIK3CA, TP53, SETD2, BRCA2, PTEN, POLE, BLM, ARID1A, ATRX, CDKN2A, MSH6, PDGFRA* were obtained from the publicly available microarray data of gene expression in the human brain from the Allen Brain Sciences Institute (www.brain-map.org). The gene expression z scores were determined based on microarray analysis of 6 adult brains that were micro-dissected into anatomically distinct brain regions (500), representing 93% of known genes that are represented by at least 2 probes. For each gene, gene expression profiles were downloaded for each available probe.

## Results

### Imaging features of pediatric diffuse midline gliomas

Five hundred gene panel testing was performed on tumor tissue of seven pediatric patients with diffuse midline glioma (n = 4) or hemispheric high-grade glioma (n = 3). Average age at surgery was 3.3 ± 1.9 years with overall survival of 9.8 ± 6.8 months. Three of the diffuse midline gliomas had the K27M mutation in the tail of histone H3 protein. One patient with diffuse midline glioma had wildtype histone H3.1 and H3.3 proteins. In addition to three hemispheric gliomas characterized in current study, tumor locations and described mutations were obtained from previously published six patients^6^. All of the patients were found to have additional mutations in genes that play a critical role in tumorigenesis. The gene mutations were different between the patients with diffuse midline gliomas and hemispheric high-grade gliomas.

While patients with diffuse midline gliomas predominantly had the histone H3 K27M mutation, additional driver mutations, such as *ACVR1* and *PPM1D* were noted in all of the patients that were analyzed with 500 gene panel testing (Table 1, Supplementary Table 1). For example, one of the patients had histone H3 K27M mutation, *PIK3CA* mutation within a highly conserved helical domain, and *PPM1D* truncation mutation (Supportinf Fig. S1). Mutations in these genes suggest that disruption of chromatin structure and gene expression mediated by histone H3 tails, combined with disruptions in the PI3K pathway and p38/MAPK pathway are critical for tumorigenesis. This 3-year-old patient presented with a solid tumor that originated within the pons with extension into the middle cerebellar peduncle. The tumor was infiltrative in appearance, had evidence of central necrosis, mild enhancement, and no reduced diffusion. On six months follow up, the tumor developed significant progression with local extension into the cerebral peduncles, temporal stem, thalamus, corpus callosum, and to the subependymal surface of the lateral ventricles. Overall survival was 6.6 months.

**Table 1:** Mutations identified within diffuse midline gliomas and hemispheric gliomas and their effect on protein structure.

Gene	Mutation	Result
PIK3CA	Q524K	Substitution of glutamine for lysine, interferes with PI3Ka_I domain responsible for substrate presentation
PIK3CA	Q546K	Substitution of glutamine for lysine, interferes with PI3Ka_I domain responsible for substrate presentation
PIK3CA	pC420R	Protein substitution of cysteine for arginine, C2_PI3K_class_I_alpha domain involved in cell processes such as cell growth, differentiation, proliferation, and motility
PPM1D	L450*	Truncation mutation, PP2C domain conserved
BCORL1	Q1043*	Truncation mutation, leads to loss of ankryn repeat domains and PUFD_like_1 domain (PCGF Ub-like fold discriminator of BCOR-like 1)
TP53	R280L	Substitution of arginine for leucine, interferes with tumor suppressor p53
TP53	pR273C	Protein substitution of arginine for cysteine, interferes with tumor suppressor function of p53
TP53	Q192H	Substitution of glutamine for histidine, interferes with tumor suppressor p53
TP53	673-1G>A	Substitution of glycine for alanine, domains conserved
TP53	R158C	Substitution of arginine for cysteine, interferes with tumor suppressor p53
TP53	N239D	Substitution of asparagine for aspartic acid, interferes with tumor suppressor p53
ACVR1	G328E	Substitution of glycine for glutamic acid, interferes with STKc_ACVR1_ALK1 includes ATP binding and substrate binding sites
ASXL1	pE566*	On protein truncation mutation, domains conserved
NF1	c7395-1G>T	On cDNA substitution of glycine for threonine, domains conserved
NF1	2851-1G>T	Substitution of glycine for threonine, domains conserved
NF1	R1362*	Truncation mutation, interferes with RasGAP domain
SETD2	T305fs	Frame shift mutation, domains conserved
SETD2	W1306*	Truncation mutation, domains conserved
SMARCB1	Deletion	22 Mb deletion on chromosome 22
NF2	Deletion	22 Mb deletion on chromosome 22
POLE	P286L	Substitution of proline for leucine, interferes with domain involved in catalytic subunit of DNA polymerase
POLE	286-1G>T	Substitution of glycine for threonine, interferes with domain involved in catalytic subunit of DNA polymerase
PTEN	K128N	Substitution of lysine for asparagine, interferes with PTEN_C2 domain responsible for membrane binding
PTEN	T277I	Substitution of threonine for isoleucine, interferes with PTEN_C2 domain responsible for membrane binding

The second patient with diffuse midline glioma was found to have mutations in histone H3.3 subtype (K27M), *BCORL1* truncation mutation, and *TP53* mutation within a highly conserved arginine location within the DNA binding domain to a hydrophobic leucine. Mutations in these genes suggest that disruptions in gene expression mediated by modifications of histone H3 tails, the TP53 pathway, and a related pathway that regulates gene expression in a histone de-acetylase related manner is required to drive tumorigenesis (Supplementary Table 1). This patient presented at 4.7 years of age with a large heterogeneously enhancing tumor within the thalamus that demonstrated minimal reduced diffusion on DWI and was associated with mass effect on the third ventricle that resulted in non-communicating hydrocephalus (Supplementary Fig. 2). On three months follow up, the tumor progressed locally to the adjacent corona radiata, subependymal surface of the ventricles, and demonstrated distal progression into the posterior fossa along the subependymal surface of the fourth ventricle. This tumor progressed quickly and patient’s overall survival after surgical intervention was only 5.6 months.

The third patient with diffuse midline glioma, H3 K27M-mutant, was also found to have *PIK3CA* mutation within a highly conserved helical domain, and an ACVR1 mutation within the kinase domain. Mutations in these genes suggest that alterations in gene expression due to mutation in histone H3 tail and disruption of PI3K pathway and Smad/PI3K/p38MAPK pathways is driving tumorigenesis (Table 1, Supplementary Table 1). This patient’s tumor presented at 1.8 years of age and originated in the cervical spinal cord demonstrating heterogeneous T2 hyperintense signal with expansion of the cord. The tumor was avidly enhancing and there was no metastatic disease within the brain on presentation (Supplementary Fig. 3). Within 5 months after diagnosis, metastatic disease to the brain via subependymal route was noted. The patient also developed significant progression of disease within lower thoracic spine with coating of the spinal cord with enhancing soft tissue mass. Brain metastatic disease demonstrated patchy FLAIR hyperintense expansile masses lining the folia of the cerebellum, subependymal surfaces of the fourth and lateral ventricles, and extension of disease into the periventricular white matter and corpus callosum. Patient’s overall survival was 17 months.

The fourth patient with diffuse midline glioma had wildtype histone H3.1 and H3.3, and on genomic sequencing, had mutations in PIK3CA conserved C2 domain, TP53 DNA binding domain, and a ASXL1 truncation mutation with loss of DNA binding domain. These mutations suggest that disruption in the PI3K pathway, TP53 pathway, and chromatin modification mediated gene expression driven by ASXL1 are driving the tumorigenesis in this tumor (Supplementary Table 1). On presentation at 4.8 years of age, this patient had a classic appearing FLAIR hyperintense infiltrating pontine glioma that demonstrated minimal enhancement and did not show reduced diffusion. The pons was expanded with near circumferential surrounding of the basilar artery without evidence of vascular compression. There was mass effect on the fourth ventricle, but no hydrocephalus was noted (Supplementary Fig. 4). Within 9 months of presentation, the tumor demonstrated extensive local metastatic spread into the cerebellar hemispheres, cerebral peduncles, thalami, sub-insular white matter and insular cortex. The overall survival was 9.7 months.

### Imaging features of pediatric hemispheric high-grade gliomas

Three of the patients in our cohort had hemispheric high-grade gliomas. One of these patients was diagnosed with neurofibromatosis 1 and developed three separate glioblastomas over the course of a year and a half (Supplementary Fig. 5). Two of the patient’s separately appearing glioblastomas within different locations (corpus callosum and temporal lobe) were sequenced and hypermutated phenotype was shown with mutations in genes *TP53*, *SETD2*, *ATRX*, *PTEN*, *POLE*, *BLM*, *ARID1A*, *APC*, *KDM6A*, *PTPN11*, and *BRCA2,* which are predominantly involved in DNA repair. Both of the tumors contained mutations in *TP53* and *NF1*, but additional mutations were different. The tumors arose in separate locations and at separate times from a background of extensive FLAIR hyperintense changes within the supratentorial and infratentorial brain parenchyma. The first tumor arose from the corpus callosum at 1.6 years of age, demonstrating FLAIR hyperintense signal and peripheral enhancement. Gross total resection of the tumor was achieved and there was no local recurrence of the tumor within the resection bed. Sixteen months after resection of the original tumor, a separate independent tumor arose from the contralateral temporal lobe and demonstrated rapid growth into a heterogeneous FLAIR hyperintense enhancing mass. Subtotal resection of the new tumor was achieved. During treatment of the temporal lobe tumor, additional tumor within a cerebellar hemisphere was also noted to arise and demonstrated rapid growth. The patient survived 23 months after diagnosis of the first tumor.

The second patient with hemispheric high-grade glioma demonstrated inactivating mutations in TP53 and PIK3CA. These mutations were similar to what is typically seen in diffuse midline gliomas but lacked a mutation in a chromatin modification related pathway. This patient was diagnosed at 2.8 years of age and was found to have a left temporal FLAIR hyperintense expansile mass that showed minimal initial enhancement and heterogeneous diffusion characteristics with regions of low and high ADC values (Supplementary Fig. 6). This tumor did not undergo gross total resection and demonstrated significant local progression within 6 months of diagnosis. On six months follow up, the tumor demonstrated heterogeneous enhancement and local spread into the adjacent basal ganglia, corpus callosum, and cerebral peduncles. The patient lived for 7 months after diagnostic biopsy.

The third patient with hemispheric high-grade glioma had wildtype histone H3 and demonstrated a hyper-mutated genotype with mutations in *ATRX*, *TP53*, *CDKN2A*, *SETD2*, *MSH6*, and *PDGFRA*. These genes are involved in the DNA repair pathway, regulation of cell cycle progression, and signaling via ERK pathway (Supplementary Tables 1, 2). This tumor was centered in the corona radiata of the parietal lobe and demonstrated avid enhancement, central necrosis, and prominent peripheral reduced diffusion. The tumor was encircled by FLAIR hyperintense signal suggesting prominent edema with microscopic infiltration of tumor into adjacent brain parenchyma. There were no follow up imaging for this patient to determine the overall survival.

### Gene expression levels based on Allen Brain Atlas gene expression atlas

Allen Brain Atlas microarray gene expression data for human brain was used to determine the normal gene expression levels of genes that were found to be mutated in diffuse midline gliomas and hemispheric high-grade gliomas. The microarray gene expression data represents gene expression in 500 anatomic locations that were micro-dissected from 6 adult normal human brains and 4 midgestational prenatal brains.

**Figure 1:**
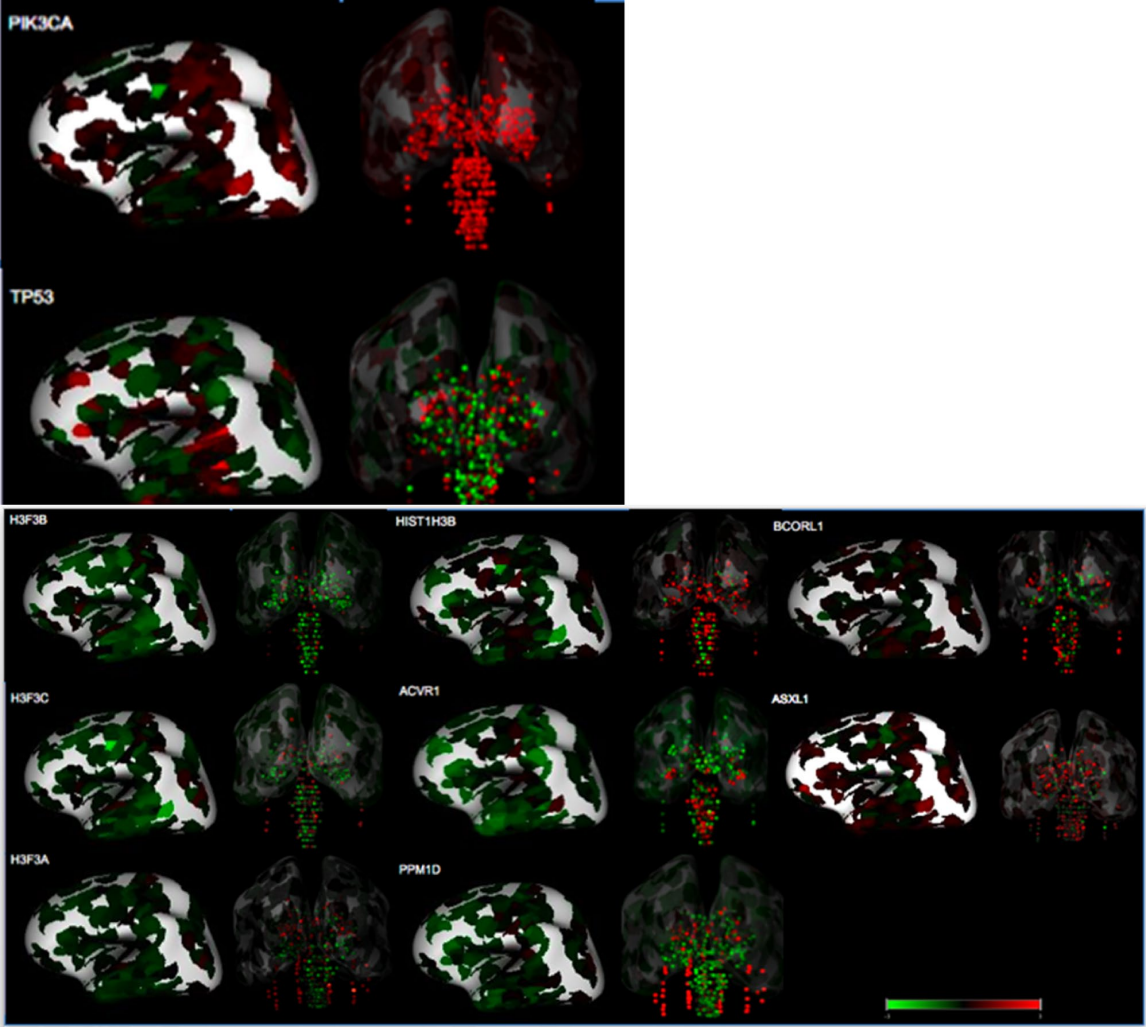
Location based human gene expression levels within the cerebral hemispheres and along the midline structures for H3F3A, H3F3B, H3F3C, HIST1H3B, ACVR1, PPM1D, BCORL1, and ASXL1. Distribution of gene expression z scores within the brain with green indicating low expression and red indicating high expression.

**Figure 2:**
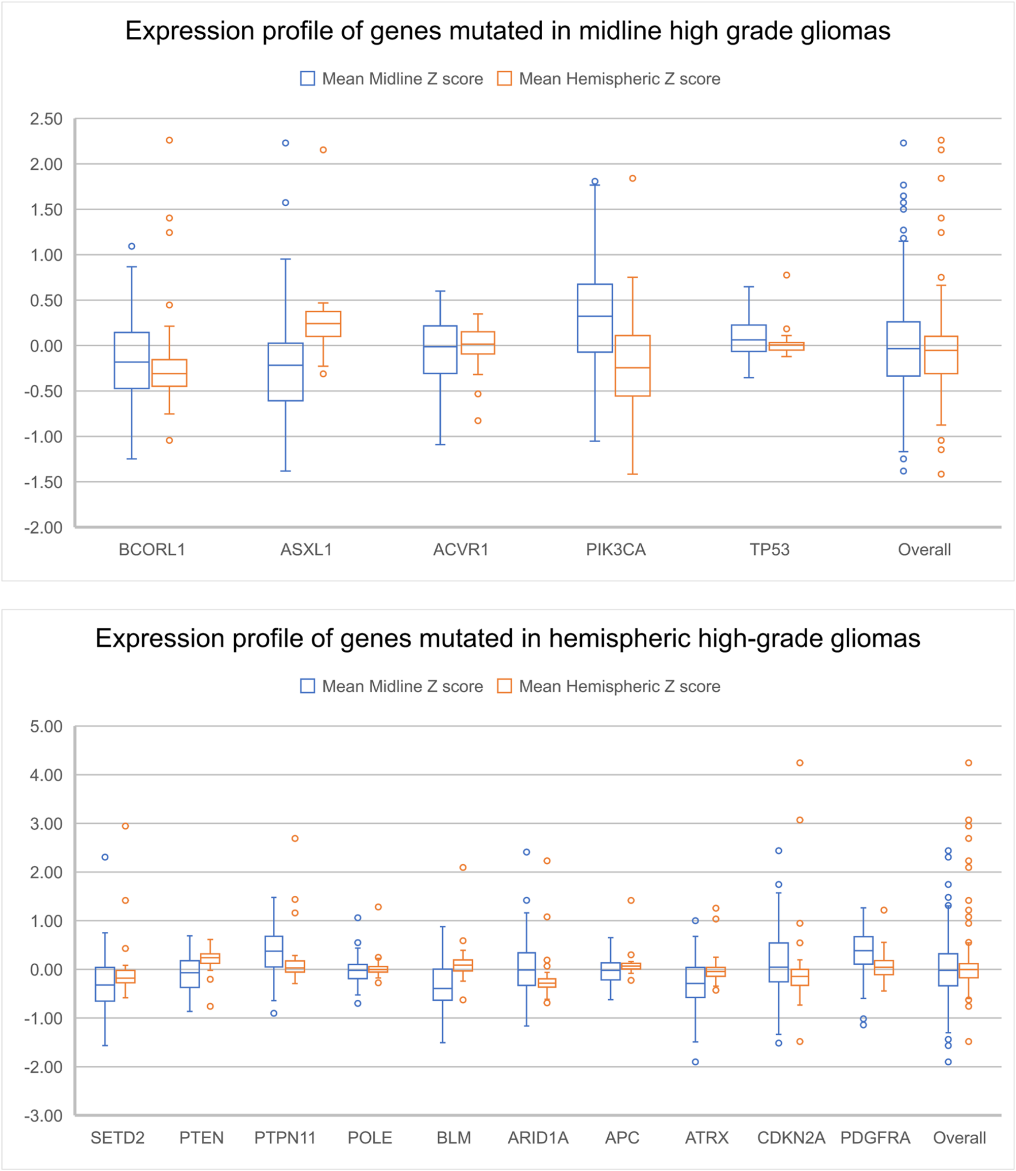
Expression profile of genes mutated in midline and hemispheric high-grade gliomas.

**Figure 3:**
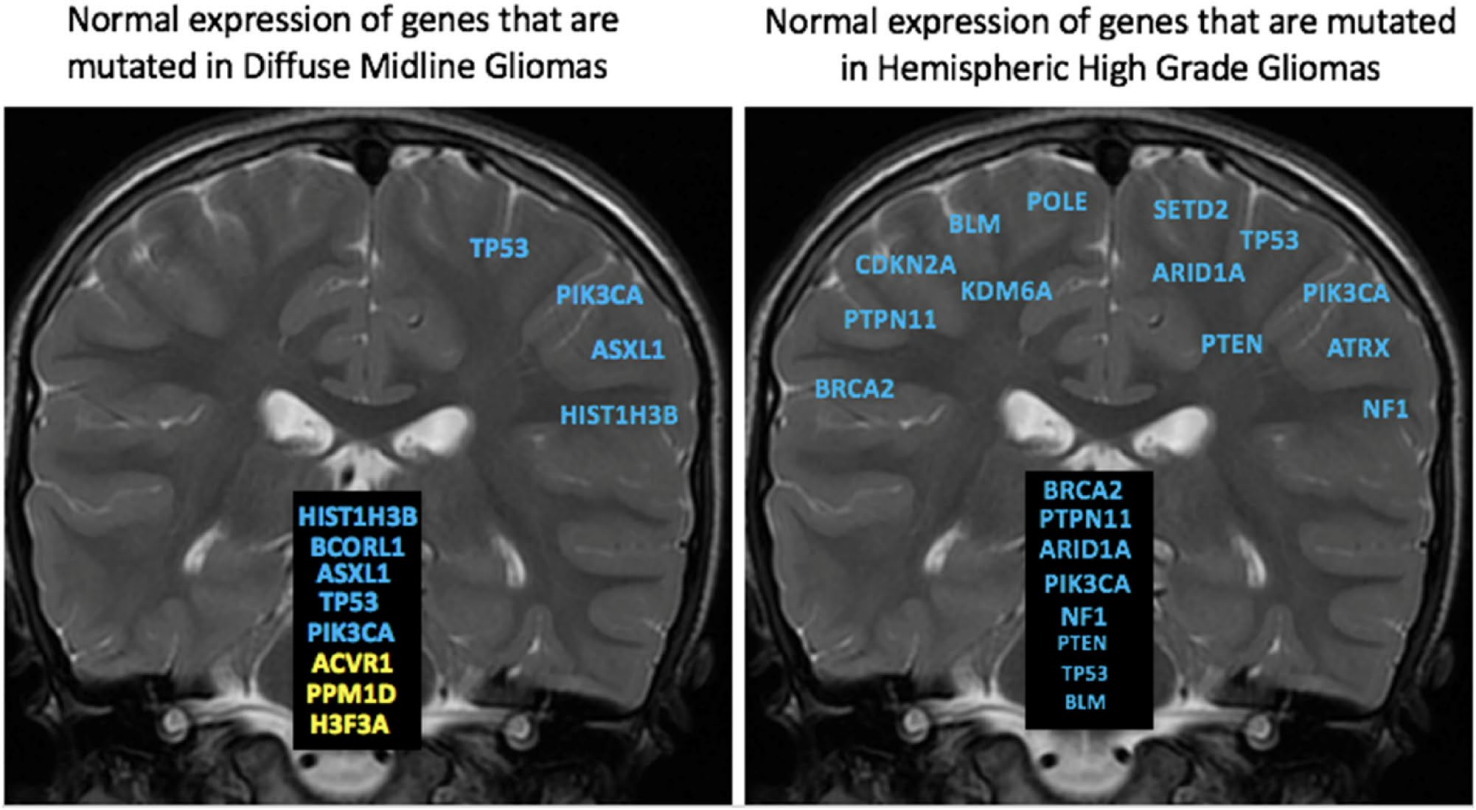
Summary of gene expression profiles of genes that are commonly mutated in diffuse midline gliomas and in hemispheric glioblastomas.

**Figure 4:**
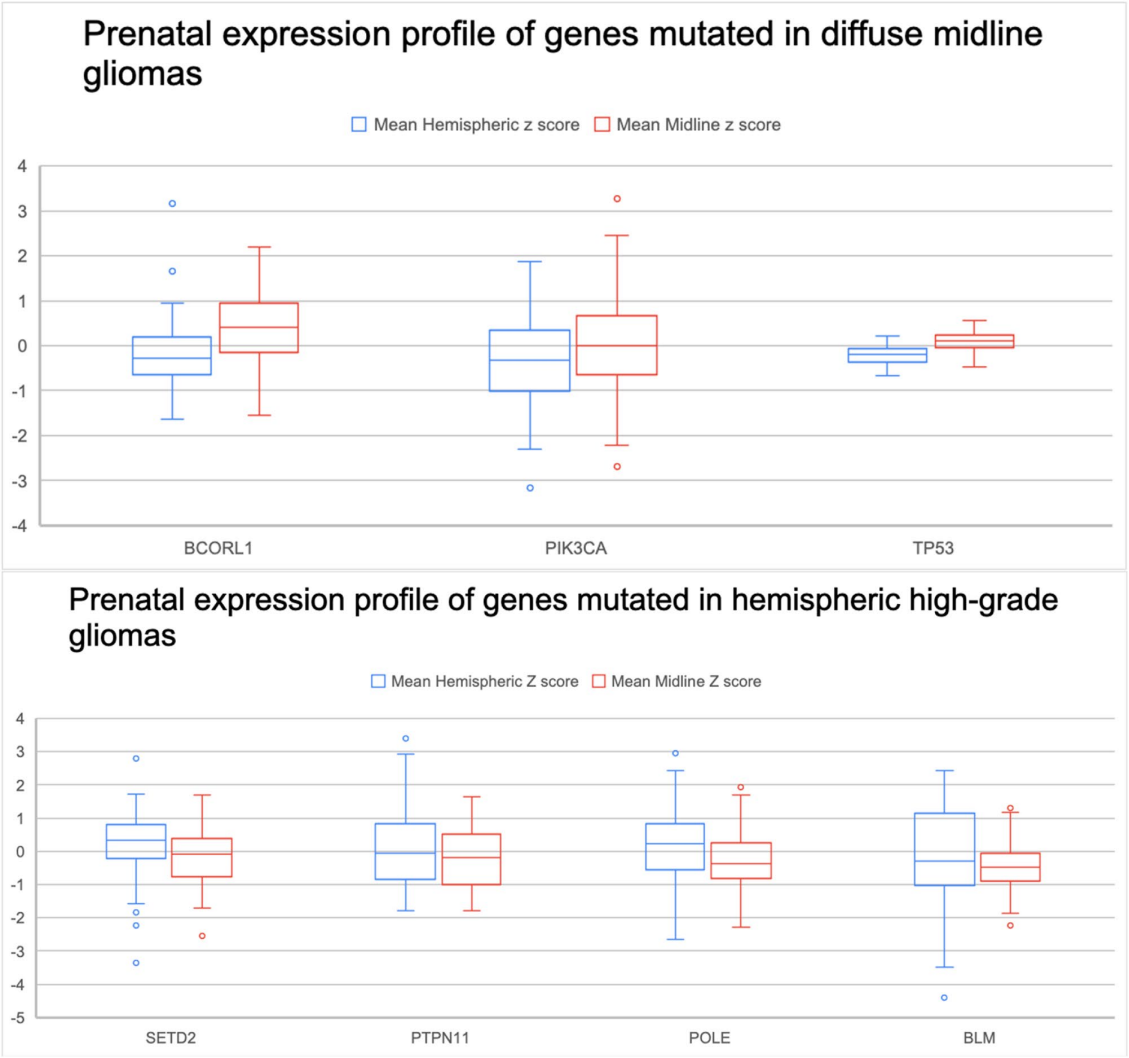
Gene expression patterns from prenatal brain atlas data.

*H3F3A*, *HIST1H3B*, *ACVR1*, *PPM1D*, *BCORL1*, and *ASXL1* genes were found to be mutated within our diffuse midline gliomas that were sequenced with 500 gene panel testing and agreed with published literature^17^. Microarray gene expression data from normal brain demonstrated that these genes are preferentially expressed along the midline including the brainstem, cerebellum, and thalamus (Figs. 1, 2, Supplementary Fig. 7). On the contrary, these genes demonstrated lower overall expression within the cerebral hemispheres (Figs. 2, 3, and 4). Two of the genes (*BCORL1* and *ASXL1*) demonstrate increased expression along both the midline and within the cerebral hemispheres. *BCORL1* gene was mutated in thalamic diffuse midline glioma in our cohort and its expression is normally elevated within the ventral thalamus, claustrum, and cingulum bundle (Fig. 1, Supplementary Fig. 7A). *PPM1D* gene was mutated in diffuse midline glioma that originated in superior and middle cerebellar peduncle, cerebral peduncle, and pons. Normally elevated expression of *PPM1D* gene is found within the thalamus, oculomotor nuclear complex of the midbrain, which is the location where tumor originated, globose nucleus, nucleus subceruleus, superior olivary complex, and central medullary reticular group (Figs. 2, 3, and 4, Supplementary Fig. 7B). *ASXL1* gene was mutated in diffuse midline glioma that originated within the pons. Normally elevated expression of *ASXL1* gene was found within superior olivary complex of the pons, medulla’s central glial substance and cuneate nucleus, rostral group of the interlaminar nuclei of the thalamus, and additional nuclei within the thalamus (Figs. 2, 3, and 4, Supplementary Fig. 7C). *ACVR1* gene mutation was found within diffuse midline glioma that originated within the cervical spine but can also be see in other midline gliomas. There was increased expression of *ACVR1* in multiple midline locations that included the thalamus and pons (Figs. 2, 3, and 4, Supplementary Fig. 7D). *PIK3CA* gene was mutated in tumors found within superior and middle cerebellar peduncle, cerebral peduncle, pons, cervical spine, temporal lobe, and insula. Elevated gene expression was also found within these anatomic regions including midbrain, pons, temporal lobe, and claustrum (Figs. 2, 3, and 4, Supplementary Fig. 7E). *TP53* gene was mutated in tumors that were found in pons, thalamus, corpus callosum, temporal lobe, and insula. Elevated gene expression was also found within these anatomic regions including thalamus, pons, temporal lobe, and claustrum (Figs. 2, 3, and 4, Supplementary Fig. 7F).

In patients who had hemispheric high-grade gliomas, there were several characteristic imaging features. These tumors were primarily aggressive locally with prominent infiltrative spread best appreciated on FLAIR MR imaging. One of the patients had gross total resection of one of the tumors with distant recurrence. These tumors were predominantly hypermutated, with one of the patients demonstrating mutations in up to 12 genes. Some of these genes were mutated at different locations and recurred in the second tumor that grew at a separate location. When looking at gene expression of these genes in normal brains, they were predominantly expressed in the cerebral hemispheres with only some of the genes demonstrating strong expression along the midline structures and along the hemispheres together (Figs. 2, 3, and 4, Supplementary Fig. 8A). *SETD2* gene was mutated in tumors originating from the corpus callosum and temporal lobe. Normal elevated expression of *SETD2* was found in corpus callosum and cingulate gyrus. There was decreased expression of *SETD2* within the temporal lobe (Supplementary Fig. 8A). *PTEN* gene was mutated in tumor originating within the temporal lobe and in none of the diffuse midline gliomas. There were elevated expression levels of *PTEN* within the temporal lobe (Figs. 2, 3, and 4, Supplementary Fig. 8B). *PTPN11* gene was mutated within a tumor that originated within corpus callosum and had highest gene expression in the corpus callosum and cingulum bundle (Figs. 2, 3, and 4, Supplementary Fig. 8C). *KDM6A* gene was mutated within a tumor that originated within corpus callosum with similar gene expression pattern being elevated within the midline structures of corpus callosum, cingulum bundle, and thalamus (Figs. 2, 3, and 4, Supplementary Fig. 8D). *POLE*, *BLM, ARID1A* and *APC* genes were mutated within a tumor that originated within the temporal lobe, but for this gene the highest expression was in the midline structures as opposed to temporal lobe (Figs. 2, 3, and 4, Supplementary Fig. 8E–H). *ATRX* gene was mutated within a tumor that originated within the white matter of the parietal lobe, but its expression within the parietal lobe was not elevated (Supplementary Fig. 8I). *CDKN2A* gene was mutated within a tumor that originated within the white matter of the parietal lobe, with its expression being highest within the cingulum bundle, claustrum, thalamus, and pons (Supplementary Fig. 8J). *MSH6* gene was mutated within a tumor that originated within the white matter of the parietal lobe with highest gene expression of being seen in corpus callosum, cingulum bundle, and thalamus (Supplementary Fig. 8K). *PDGFRA* gene was mutated within a tumor that originated within the white matter of the parietal lobe with the predominant normal gene expression being found in pons, thalamus, and claustrum (Supplementary Fig. 8L). These findings suggest that not all of the genes that are mutated in the hemispheric tumors strictly have highest expression within the hemispheric structures, but when looking at overall expression patterns they still have higher expression along hemispheric structures (Figs. 2, 3, and 4).

Comparing endogenous gene expression z scores between genes that are commonly mutated in high grade midline gliomas versus high grade hemispheric gliomas demonstrates a difference in gene expression patterns in both adult and developmental human datasets (Figs. 2, 3, and 4). In the developmental brain dataset, the differences in hemispheric expression of PTEN and APC are not as elevated as seen in adults (Figs. 2 and 4). We noted the expression patterns of histone genes *HIST1H3B* and *H3F3A* (Supplementary Table 20, Supplementary Table 21, Supplementary Fig. 8M, Supplementary Fig. 8N). *BCORL1, PPM1D, PIK3CA*, and *TP53* appear to have higher expression z scores along the midline structures as compared to hemispheric structures. On the other hand, *SETD2, PTEN, PTPN11, POLE, BLM*, and *APC* appear to have higher expression z scores along the hemispheric structures as compared to midline structures.

## Discussion

We report the imaging features of pediatric high-degrade gliomas that are located along the midline and within the cerebral hemispheres and that had targeted next-generation gene sequencing analysis. We found that in our diffuse midline gliomas, additional driver mutations are always present, regardless of histone H3 mutation status. This agrees with published literature that multiple mutations are found in these tumors^17,18^. The genes that are mutated in the diffuse midline gliomas contribute to coordination of three distinct pathways. A chromatin modification alteration was always present in these tumors and is represented by modification of histone H3 tails or by presence of mutation in a chromatin modifying gene, such as ASXL1. In addition to alteration of chromatin modification, mutations in PI3K pathway are also found. One patient did not have PI3K mutation, but did have alteration in BCORL1, which is involved in histone deacetylation and has been shown to be involved in cross-talk with PI3K pathway^19^. In addition to chromatin modification and PI3K pathway alterations, mutations in TP53 pathway were also noted in our diffuse midline gliomas. In cases where TP53 was not mutated, genes involved in MAPK pathway, which is involved in crosstalk with TP53 pathway, were mutated^20^. This suggests that drivers of tumorigenesis in diffuse midline gliomas need to combine three distinct pathways—chromatin modification alteration, PI3K pathway, and p53 mediated MAPK cross-talk (Supplementary Fig. 9).

While the genes mutated in diffuse midline gliomas are normally expressed along the midline structures of the brain (Figs. 2, 3, and 4), in patients with hemispheric glioblastomas with wildtype histone H3, hypermutated genomes with mutations in multiple genes that play a critical role as tumor suppressors and DNA repair were seen in one of our patients and have been described in the literature (Supplementary Table 22)^6,8^. These genes are found to be normally expressed either along the cerebral hemisphere white matter or along both the cerebral hemispheres and midline structures (Figs. 2, 3, and 4). The differences in gene expression z-scores are very small, which is expected in normal brain tissues. The confirmation of the patterns that we see in adults with developmental brain data supports our original hypothesis.

*TP53* and *PIK3CA* were found to be mutated in both midline and hemispheric high-grade gliomas. The exact *TP53* mutations were different between the different patients that were analyzed, but involved critical function of DNA binding domains. Two of the diffuse midline gliomas and all three of the hemispheric gliomas had mutations in TP53. In addition, three of the diffuse midline gliomas and one of the hemispheric gliomas had mutation in PIK3CA, indicating a critical role of the PI3 kinase pathway in tumorigenesis. Normal gene expression analysis of these genes in humans demonstrates prominent expression in both the hemispheres and midline structures suggesting they are important in normal function. Expression of these genes within specific parts of the brain may predispose them to become mutated in the setting of open actively transcribed chromatin and may play a role in specific selection of drivers of tumorigenesis by tumors within specific locations of the brain.

There a number of limitations in our paper including small sample size and our cohort not including patients with mutations in *IDH1/2* genes, which are common in adult hemispheric gliomas. Even though our cohort size is small, the genes that were identified on targeted next generation sequencing agree with published literature on more comprehensive datasets^17^. In addition, gene expression profiles in humans ranging from 24 to 57 years of age were analyzed in Allen Brain Atlas Database, which we confirmed on the developing brain database within Allen Brain Atlas. Future studies should include gene expression profiles from pediatric patients as this data becomes more available. In addition, clonality of mutations within the tumors was not evaluated in the current study, which is an important area of research for future analysis^21,22^. In addition to regional microdissection-based analysis of gene expression, focused single cell analysis within specific regions of the brain of the genes that are known to be mutated in pediatric brain tumors will provide critical information on etiology of tumorigenesis in midline versus hemispheric pediatric gliomas.

In conclusion, we characterize the imaging localization of tumors in pediatric patients which had targeted next-generation sequencing. We found that in diffuse midline gliomas three major pathways were mutated in every patient—chromatin modification, PI3 kinase pathway, and TP53 pathway. Among the hemispheric high-grade glioma patients, we had one patient with hypermutated glioblastomas and another patient with defects in the TP53 pathway and PI3 kinase pathway. To increase the number of our patients, we performed a multi-institutional study, which included patients with 4 additional hemispheric high-grade gliomas, 1 cerebellar hemisphere glioma, and 6 pontine diffuse midline gliomas (Table 2). Gene mutations in these patients followed similar trend, with TP53 mutations being found in hemispheric high-grade gliomas, while histone H3 K27M mutation being found in diffuse midline gliomas. The limitation of added patients from a second institution is that sequencing analysis in these tumors was limited to 50 gene panel, which does not include many of the driver mutations found in pediatric brain tumors. On analysis of temporal expression profile of normal gene expression in the brain, we found that genes that were mutated in patients with midline gliomas, were usually had normal higher expression in the anatomic locations where these tumors arise. This suggests that normal gene expression profile and brain microenvironment affects predisposition to mutagenesis and may play a role in defining driver mutations in tumors that arise from different parts of the brain.

**Table 2:** Mutations identified in individual pediatric brain tumors.

Patient	Pathological diagnosis	Mutations	Tumor origin
1	Diffuse midline glioma	H3 K27M, PIK3CA, PPM1D	Pons
2	Diffuse midline glioma	H3 K27M, BCORL1, TP53, PDGFRA	Thalamus
3	Diffuse midline glioma	H3 K27M, PIK3CA, ACVR1	Cervical spinal cord
4	Diffuse midline glioma	PIK3CA, TP53, ASLX1	Pons
5	Glioblastoma	TP53, NF1, SETD2, ATRX, KDM6, PTPN11, BRCA2	Corpus collosum
5	Glioblastoma	TP53, NF1, PTEN, SETD2, POLE, BLM, ARID1A, APC	Temporal lobe
6	Astrocytoma, grade III	TP53, PIK3CA	Temporal lobe
7	Glioblastoma	ATRX, TP53, CDKN2A, SETD2, MSH6, PDGFRA	Parietal lobe
8	Glioblastoma	SMARCB1, NF2	Frontal lobe
9	Glioblastoma	TP53, EGFR	Temporal lobe
10	Anaplastic astrocytoma	IDH1, TP53	Frontal lobe
11	Glioblastoma	No histone H3 or IDH mutation	Cerebellar hemisphere
12	Glioblastoma	IDH1, TP53	Frontal lobe
13	Glioblastoma	TP53	Frontal lobe
14	Diffuse midline glioma	Histone H3 K27M, TP53	Pons
15	Diffuse midline glioma	PDGFRA, no histone H3 mutation	Pons
16	Diffuse midline glioma	Histone H3 K27M, TP53, PTEN	Pons
17	Diffuse midline glioma	Histone H3 K28M, TERT	Pons
18*	Diffuse midline glioma	Histone H3 K27M	Pons
19*	Diffuse midline glioma	Histone H3 K27M	Pons

Patients 1–8 underwent 500 gene panel mutation analysis. Patients 9–16 underwent either 50 gene panel mutation analysis. Patient 17 underwent Foundation One analysis. Patients 18 and 19 underwent standard pathologic analysis with immunohistochemistry stains for histone H3 K27M mutation. 50 gene panel analysis includes PIK3CA but not ACVR1 mutation analysis.

## Supplementary Information

Supplementary Information.

## References

[1] Castel D, Philippe C, Kergrohen T (2018). Transcriptomic and epigenetic profiling of 'diffuse midline gliomas, H3 K27M-mutant' discriminate two subgroups based on the type of histone H3 mutated and not supratentorial or infratentorial location. Acta Neuropathol. Commun..

[2] Ezaki T, Sasaki H, Hirose Y, Miwa T, Yoshida K, Kawase T (2011). Molecular characteristics of pediatric non-ependymal, nonpilocytic gliomas associated with resistance to temozolomide. Mol. Med. Rep..

[3] Jones DTW, Banito A, Grunewald TGP (2019). Molecular characteristics and therapeutic vulnerabilities across paediatric solid tumours. Nat. Rev. Cancer.

[4] Nikbakht H, Panditharatna E, Mikael LG (2016). Spatial and temporal homogeneity of driver mutations in diffuse intrinsic pontine glioma. Nat. Commun..

[5] Himes BT, Zhang L, Daniels DJ (2019). Treatment strategies in diffuse midline gliomas with the H3K27M mutation: The role of convection-enhanced delivery in overcoming anatomic challenges. Front. Oncol..

[6] Kline CN, Joseph NM, Grenert JP (2017). Targeted next-generation sequencing of pediatric neuro-oncology patients improves diagnosis, identifies pathogenic germline mutations, and directs targeted therapy. Neuro Oncol..

[7] Williams JR, Young CC, Vitanza NA (2020). Progress in diffuse intrinsic pontine glioma: Advocating for stereotactic biopsy in the standard of care. Neurosurg. Focus.

[8] Coleman C, Stoller S, Grotzer M, Stucklin AG, Nazarian J, Mueller S (2020). Pediatric hemispheric high-grade glioma: Targeting the future. Cancer Metast. Rev..

[9] Johnson A, Severson E, Gay L (2017). Comprehensive genomic profiling of 282 pediatric low- and high-grade gliomas reveals genomic drivers, tumor mutational burden, and hypermutation signatures. Oncologist.

[10] Chamdine O, Gajjar A (2014). Molecular characteristics of pediatric high-grade gliomas. CNS Oncol..

[11] Rodriguez Gutierrez D, Jones C, Varlet P (2020). radiological evaluation of newly diagnosed non-brainstem pediatric high-grade glioma in the HERBY phase II trial. Clin. Cancer Res..

[12] Aboian MS, Solomon DA, Felton E (2017). Imaging characteristics of pediatric diffuse midline gliomas with histone H3 K27M mutation. AJNR Am. J. Neuroradiol..

[13] Bozkurt SU, Dagcinar A, Tanrikulu B (2018). Significance of H3K27M mutation with specific histomorphological features and associated molecular alterations in pediatric high-grade glial tumors. Childs Nerv. Syst..

[14] Broniscer A, Hwang SN, Chamdine O (2018). Bithalamic gliomas may be molecularly distinct from their unilateral high-grade counterparts. Brain Pathol..

[15] El-Ayadi M, Ansari M, Sturm D (2017). High-grade glioma in very young children: A rare and particular patient population. Oncotarget.

[16] Makova KD, Hardison RC (2015). The effects of chromatin organization on variation in mutation rates in the genome. Nat. Rev. Genet..

[17] Mackay A, Burford A, Carvalho D (2017). Integrated molecular meta-analysis of 1,000 pediatric high-grade and diffuse intrinsic pontine glioma. Cancer Cell.

[18] Georgescu MM, Islam MZ, Li Y (2020). Global activation of oncogenic pathways underlies therapy resistance in diffuse midline glioma. Acta Neuropathol. Commun..

[19] Citro S, Miccolo C, Meloni L, Chiocca S (2015). PI3K/mTOR mediate mitogen-dependent HDAC1 phosphorylation in breast cancer: A novel regulation of estrogen receptor expression. J. Mol. Cell Biol..

[20] Stramucci L, Pranteda A, Bossi G (2018). Insights of crosstalk between p53 protein and the MKK3/MKK6/p38 MAPK signaling pathway in cancer. Cancers.

[21] Chowell D, Napier J, Gupta R, Anderson KS, Maley CC, Sayres MAW (2018). Modeling the subclonal evolution of cancer cell populations. Cancer Res..

[22] Salichos L, Meyerson W, Warrell J, Gerstein M (2020). Estimating growth patterns and driver effects in tumor evolution from individual samples. Nat. Commun..

