# Author Correction: Topographic correlates of driver mutations and endogenous gene expression in pediatric diffuse midline gliomas and hemispheric high-grade gliomas

**DOI:** 10.1038/s41598-021-96015-1

**Published:** 2021-08-11

**Authors:** Eve Kazarian, Asher Marks, Jin Cui, Armine Darbinyan, Elizabeth Tong, Sabine Mueller, Soonmee Cha, Mariam S. Aboian

**Affiliations:** 1 Department of Radiology & Biomedical Imaging, Yale School of Medicine, New Haven, CT USA; 2 Department of Pediatric Hematology & Oncology, Yale School of Medicine, New Haven, CT USA; 3 Department of Neuropathology, Yale School of Medicine, New Haven, CT USA; 4 Department of Radiology, University of California, San Francisco, San Francisco, USA; 5 Division of Pediatric Hematology & Oncology, Department of Pediatrics, University of California, San Francisco, San Francisco, USA; 6 Department of Neurological Surgery, University of California, San Francisco, San Francisco, USA; 7 Department of Neurology, University of California, San Francisco, San Francisco, USA

Correction to: *Scientific Reports* 10.1038/s41598-021-92943-0, published online 13 July 2021

The Funding section in the original version of this Article was omitted. The Funding section now reads:

“American Society of Neuroradiology Fellow Award 2018 (MSA). This publication was made possible by KL2 TR001862 from the National Center for Advancing Translational Science (NCATS), components of the National Institutes of Health (NIH), and NIH roadmap for Medical Research. Its contents are solely the responsibility of the authors and do not necessarily represent the official view of NIH.”

The original Article has been corrected.

