# Supplementary material for: Topographic correlates of driver mutations and endogenous gene expression in pediatric diffuse midline gliomas and hemispheric high-grade gliomas
Source: Sci Rep. 2021 Jul 13;11:14377. doi: 10.1038/s41598-021-92943-0 (PMC8277861; doi:10.1038/s41598-021-92943-0)
Supplement: Supplementary file 1 — Supplementary Information. [file 41598_2021_92943_MOESM1_ESM.docx]

**Supplementary Table 1.** Tumor locations of origin for genetic mutations, organized by glioma type.

|  | **Diffuse midline** | **Hemispheric high-grade** | **Pathway** |
| --- | --- | --- | --- |
| **H3 K27M** | Pons, thalamus, cervical spine |  | Chromatin modification |
| **PPM1D** | Superior and middle cerebellar peduncle, cerebral peduncle, pons |  | Expression induced by p53, negatively regulates p38/MAPK pathway |
| **ASXL1** | Pons |  | Chromatin binding protein that can disrupt chromatin modifications and can result in alteration of gene expression |
| **BCORL1** | Thalamus |  | Transcriptional co-repressor and interacts with histone de-acetylases |
| **ACVR1** | Cervical spine |  | Transmembrane protein similar to TGFbeta, implicated in Smad pathway, p38/MAPK pathway, and PI3K pathway |
| **PIK3CA** | Superior and middle cerebellar peduncle, cerebral peduncle, pons, cervical spine | Temporal lobe, insula | Integral part of PI3K pathway |
| **TP53** | Pons, thalamus | Corpus collosum, temporal lobe, insula | Tumor suppressor, integral response to DNA damage |
| **SETD2** |  | Corpus collosum, temporal lobe | Histone methyltransferase that is specific for lysine 36 on histone tails |
| **PTPN11** |  | Corpus collosum | Plays a role in RAS/MAPK pathway |
| **KDM6A** |  | Corpus collosum | Histone modification, lysine specific demethylase |
| **BRCA2** |  | Corpus collosum | Tumor suppressor, DNA repair |
| **PTEN** |  | Temporal lobe | Tumor suppressor, phosphatase that antagonizes PI3K-AKT/PKB pathway |
| **POLE** |  | Temporal lobe | Catalytic subunit of DNA polymerase epsilon, DNA repair |
| **BLM** |  | Temporal lobe | DNA helicase, involved in DNA repair |
| **ARID1A** |  | Temporal lobe | SWI/SNF family, chromatin modification |
| **APC** |  | Temporal lobe | Tumor suppressor, negative regulation of Wnt signaling |
| **ATRX** |  | Corona radiata of parietal lobe | SWI/SNF family, chromatin modification |
| **CDKN2A** |  | Corona radiata of parietal lobe | Tumor suppressor, regulation of cell cycle G1 progression, stabilizes p53 |
| **MSH6** |  | Corona radiata of parietal lobe | Mismatch repair, interacts with histone tails |
| **PDGFRA** |  | Corona radiata of parietal lobe | Transmembrane protein, tyrosine kinase, MAPK pathways, STAT activation |

**Supplementary Table 2:** Mutations identified in individual pediatric brain tumors. Patients 1-8 underwent 500 gene panel mutation analysis. Patients 9-16 underwent either 50 gene panel mutation analysis. Patient 17 underwent Foundation One analysis. Patients 18 and 19 underwent standard pathologic analysis with immunohistochemistry stains for histone H3 K27M mutation. 50 gene panel analysis includes PIK3CA but not ACVR1 mutation analysis.

| **Patient** | **Pathological Diagnosis** | **Mutations** | **Tumor Origin** |
| --- | --- | --- | --- |
| 1 | Diffuse midline glioma | H3 K27M, PIK3CA, PPM1D | Pons |
| 2 | Diffuse midline glioma | H3 K27M, BCORL1, TP53, PDGFRA | Thalamus |
| 3 | Diffuse midline glioma | H3 K27M, PIK3CA, ACVR1 | Cervical spinal cord |
| 4 | Diffuse midline glioma | PIK3CA, TP53, ASLX1 | Pons |
| 5 | Glioblastoma | TP53, NF1, SETD2, ATRX, KDM6, PTPN11, BRCA2 | Corpus collosum |
| 5 | Glioblastoma | TP53, NF1, PTEN, SETD2, POLE, BLM, ARID1A, APC | Temporal lobe |
| 6 | Astrocytoma, Grade III | TP53, PIK3CA | Temporal lobe |
| 7 | Glioblastoma | ATRX, TP53, CDKN2A, SETD2, MSH6, PDGFRA | Parietal lobe |
| 8 | Glioblastoma | SMARCB1, NF2 | Frontal lobe |
| 9 | Glioblastoma | TP53, EGFR | Temporal lobe |
| 10 | Anaplastic astrocytoma | IDH1, TP53 | Frontal lobe |
| 11 | Glioblastoma | No histone H3 or IDH mutation | Cerebellar hemisphere |
| 12 | Glioblastoma | IDH1, TP53 | Frontal lobe |
| 13 | Glioblastoma | TP53 | Frontal lobe |
| 14 | Diffuse Midline Glioma | Histone H3 K27M, TP53 | Pons |
| 15 | Diffuse Midline Glioma | PDGFRA, no histone H3 mutation | Pons |
| 16 | Diffuse Midline Glioma | Histone H3 K27M, TP53, PTEN | Pons |
| 17 | Diffuse Midline Glioma | Histone H3 K28M, TERT | Pons |
| 18* | Diffuse Midline Glioma | Histone H3 K27M | Pons |
| 19* | Diffuse Midline Glioma | Histone H3 K27M | Pons |

**Supplementary Table 3**: Quantitation of z scores of gene expression: *BCORL1.*

| **Location** | **Z score** |
| --- | --- |
| cingulum bundle | 0.44635 |
| claustrum | 1.244225 |
| corpus callosum | -0.09238 |
| dorsal thalamus | -0.10201 |
| frontal lobe | -0.30858 |
| hypothalamus | -0.42971 |
| insula | -0.21643 |
| occipital lobe | -0.19836 |
| parietal lobe | -0.20181 |
| temporal lobe | -0.26327 |
| ventral thalamus | 0.867475 |

| **Location** | **Z score** |
| --- | --- |
| basal part of pons | -0.44381 |
| central medullary reticular group | 0.438813 |
| cingulum bundle | 2.2613 |
| claustrum | 0.032542 |
| corpus callosum | 1.404483 |
| dorsal thalamus | -0.26558 |
| epithalamus | -0.34919 |
| frontal lobe | -0.44413 |
| globose nucleus | 0.183663 |
| hypothalamus | -0.65031 |
| insula | -0.48871 |
| mesencephalon | -0.34891 |
| nucleus subceruleus | 0.2204 |
| occipital lobe | -0.19086 |
| parietal lobe | -0.28825 |
| pontine tegmentum | -0.19762 |
| subthalamus | 0.353275 |
| superior olivary complex | 0.43285 |
| ventral thalamus | 1.109458 |

**Supplementary Table 4:** Quantitation of z scores of gene expression: *PPM1D*.

**Supplementary Table 5:** Quantitation of z scores of gene expression: *ASXL1*.

| **Location** | **Z score** |
| --- | --- |
| basal part of pons | -0.65129 |
| central glial substance | 2.230688 |
| cingulum bundle | 4.12795 |
| claustrum | 0.326742 |
| corpus callosum | 2.154225 |
| dorsal thalamus | -0.11818 |
| epithalamus | -0.32298 |
| frontal lobe | 0.161095 |
| hypothalamus | -0.22672 |
| insula | 0.253658 |
| mesencephalon | -0.29114 |
| occipital lobe | 0.30786 |
| parietal lobe | 0.290413 |
| pontine tegmentum | -0.37766 |
| rostral group of intralaminar nuclei | -0.65291 |
| subthalamus | 0.853383 |
| superior olivary complex | 0.46478 |
| ventral thalamus | 1.574133 |

**Supplementary Table 6:** Quantitation of z scores of gene expression: *ACVR1*.

| **Location** | **Z score** |
| --- | --- |
| basal part of pons | 0.291288 |
| cingulum bundle | 0.2678 |
| claustrum | -0.06308 |
| corpus callosum | -0.82656 |
| dorsal thalamus | 0.31546 |
| frontal lobe | -0.04651 |
| hypothalamus | -0.54621 |
| insula | -0.045 |
| mesencephalon | -0.07694 |
| occipital lobe | 0.240557 |
| parietal lobe | 0.166328 |
| pontine tegmentum | -0.03915 |
| temporal lobe | 0.055064 |
| ventral thalamus | -0.01795 |

| **Location** | **Z score** |
| --- | --- |
| basal part of pons | 0.709563 |
| cingulum bundle | 1.8411 |
| claustrum | -0.75278 |
| corpus callosum | -1.40146 |
| frontal lobe | -0.33063 |
| insula | -0.6793 |
| mesencephalon | 0.176911 |
| occipital lobe | 0.46302 |
| parietal lobe | 0.33903 |
| pontine tegmentum | 0.669242 |
| temporal lobe | -0.2811 |

| **Location** | **Z score** |
| --- | --- |
| basal part of pons | -0.13458 |
| cingulum bundle | 0.776308 |
| claustrum | 0.242169 |
| corpus callosum | 0.042956 |
| dorsal thalamus | 0.017921 |
| epithalamus | 0.035546 |
| frontal lobe | -0.01891 |
| hypothalamus | 0.056415 |
| insula | -0.0267 |
| occipital lobe | -0.0677 |
| parietal lobe | 0.001116 |
| pontine tegmentum | 0.119155 |
| subthalamus | 0.479372 |
| temporal lobe | 0.047873 |
| ventral thalamus | 0.646798 |

**Supplementary Table 7:** Quantitation of z scores of gene expression: *PIK3CA*.

**Supplementary Table 8:** Quantitation of z scores of gene expression: *TP53*.

**Supplementary Table 9:** Quantitation of z scores of gene expression: *PTEN*.

| **Location** | **Z score** |
| --- | --- |
| basal part of pons | 0.376375 |
| cingulum bundle | 0.613675 |
| claustrum | 0.492767 |
| corpus callosum | -0.75975 |
| dorsal thalamus | 0.163732 |
| frontal lobe | 0.176179 |
| hypothalamus | -0.37153 |
| insula | 0.194078 |
| mesencephalon | -0.13633 |
| occipital lobe | 0.268689 |
| parietal lobe | 0.341577 |
| pontine tegmentum | 0.024124 |
| subthalamus | 0.131044 |
| temporal lobe | 0.196377 |
| ventral thalamus | 0.07249 |

**Supplementary Table 10:** Quantitation of z scores of gene expression: *PTPN11*.

| **Location** | **Z score** |
| --- | --- |
| basal part of pons | -0.55317 |
| cingulum bundle | 2.689917 |
| claustrum | 1.160817 |
| corpus callosum | 1.436506 |
| dorsal thalamus | 0.082413 |
| epithalamus | 0.450571 |
| frontal lobe | 0.00321 |
| hypothalamus | -0.17458 |
| insula | -0.12513 |
| mesencephalon | 0.643621 |
| occipital lobe | 0.07451 |
| parietal lobe | 0.14443 |
| pontine tegmentum | 0.374466 |
| ventral thalamus | 1.400517 |

| **Location** | **Z score** |
| --- | --- |
| basal part of pons | -0.36029 |
| cingulum bundle | 3.1744 |
| claustrum | 0.582875 |
| corpus callosum | 2.203483 |
| dorsal thalamus | -0.15435 |
| frontal lobe | -0.30285 |
| hypothalamus | -0.36876 |
| insula | -0.28375 |
| mesencephalon | -0.11707 |
| occipital lobe | -0.25022 |
| parietal lobe | -0.29536 |
| pontine tegmentum | 0.144873 |
| subthalamus | 0.970346 |
| temporal lobe | -0.22991 |
| ventral thalamus | 1.675258 |

**Supplementary Table 11:** Quantitation of z scores of gene expression: *KDM6A*.

| **Location** | **Z score** |
| --- | --- |
| basal part of pons | 0.081573 |
| cingulum bundle | 1.282285 |
| claustrum | 0.22534 |
| corpus callosum | 0.098253 |
| dorsal thalamus | -0.019 |
| frontal lobe | -0.03992 |
| hypothalamus | -0.38675 |
| insula | -0.04416 |
| mesencephalon | -0.06577 |
| occipital lobe | -0.07225 |
| parietal lobe | 0.013924 |
| pontine tegmentum | 0.047972 |
| subthalamus | 0.403401 |
| temporal lobe | -0.01735 |
| ventral thalamus | 0.549186 |

**Supplementary Table 12:** Quantitation of z scores of gene expression: *POLE*.

| **Location** | **Z score** |
| --- | --- |
| basal part of pons | -0.40292 |
| cingulum bundle | 2.09352 |
| claustrum | -0.21519 |
| corpus callosum | 0.589257 |
| dorsal thalamus | -0.35547 |
| frontal lobe | 0.057967 |
| hypothalamus | -0.53393 |
| insula | -0.02854 |
| mesencephalon | -0.38733 |
| occipital lobe | 0.219589 |
| parietal lobe | 0.183028 |
| pontine tegmentum | 0.088215 |
| subthalamus | 0.07266 |
| temporal lobe | 0.111235 |
| ventral thalamus | 0.831217 |

**Supplementary Table 13:** Quantitation of z scores of gene expression: *BLM*.

| **Location** | **Z score** |
| --- | --- |
| basal part of pons | 0.065431 |
| cingulum bundle | 1.417254 |
| claustrum | 0.299091 |
| corpus callosum | 0.007477 |
| dorsal thalamus | 0.029208 |
| frontal lobe | 0.036667 |
| hypothalamus | -0.28046 |
| insula | 0.040269 |
| mesencephalon | -0.04524 |
| occipital lobe | 0.030533 |
| parietal lobe | 0.110023 |
| pontine tegmentum | 0.068823 |
| temporal lobe | 0.075107 |
| ventral thalamus | 0.650834 |

**Supplementary Table 14:** Quantitation of z scores of gene expression: *ARID1A*.

| **Location** | **Z score** |
| --- | --- |
| basal part of pons | -0.91136 |
| cingulum bundle | 2.231467 |
| claustrum | 1.079272 |
| corpus callosum | 1.108183 |
| dorsal thalamus | 0.098975 |
| frontal lobe | -0.30959 |
| hypothalamus | -0.4635 |
| insula | -0.19993 |
| mesencephalon | -0.03582 |
| occipital lobe | -0.32556 |
| parietal lobe | -0.38275 |
| pontine tegmentum | -0.0249 |
| subthalamus | 0.942644 |
| ventral thalamus | 1.108839 |

**Supplementary Table 15:** Quantitation of z scores of gene expression: *APC*.

| **Location** | **Z score** |
| --- | --- |
| basal part of pons | -0.69736 |
| cingulum bundle | 1.25842 |
| claustrum | 1.036113 |
| corpus callosum | -0.32632 |
| dorsal thalamus | -0.21627 |
| frontal lobe | -0.15371 |
| hypothalamus | -0.16887 |
| insula | 0.012348 |
| mesencephalon | -0.21057 |
| occipital lobe | 0.048311 |
| parietal lobe | -0.03784 |
| pontine tegmentum | -0.50774 |
| temporal lobe | 0.004276 |
| ventral thalamus | 0.0026 |

**Supplementary Table 16:** Quantitation of z scores of gene expression: *ATRX*.

**Supplementary Table 17:** Quantitation of z scores of gene expression: *CDKN2A*.

| **Location** | **Z score** |
| --- | --- |
| basal part of pons | 0.212652 |
| cingulum bundle | 0.946927 |
| claustrum | 0.54502 |
| corpus callosum | -0.71721 |
| dorsal thalamus | -0.03998 |
| frontal lobe | -0.03563 |
| hypothalamus | -0.15947 |
| insula | -0.06201 |
| mesencephalon | -0.03286 |
| occipital lobe | -0.09096 |
| parietal lobe | 0.001059 |
| pontine tegmentum | 0.254705 |
| temporal lobe | -0.00328 |
| ventral thalamus | 0.547344 |

| **Location** | **Z score** |
| --- | --- |
| basal part of pons | -0.12919 |
| cingulum bundle | 4.24505 |
| claustrum | 0.633558 |
| corpus callosum | 3.068683 |
| dorsal thalamus | 0.137965 |
| frontal lobe | -0.35712 |
| hypothalamus | -0.57927 |
| insula | -0.4332 |
| mesencephalon | 0.045462 |
| occipital lobe | -0.32749 |
| parietal lobe | -0.27003 |
| pontine tegmentum | 0.47367 |
| temporal lobe | -0.2823 |
| ventral thalamus | 2.438258 |

**Supplementary Table 18:** Quantitation of z scores of gene expression: *MSH6*.

| **Location** | **Z score** |
| --- | --- |
| basal part of pons | -0.01102 |
| claustrum | 0.319896 |
| dorsal thalamus | 0.133277 |
| epithalamus | -0.65241 |
| frontal lobe | 0.033803 |
| hypothalamus | 0.141591 |
| insula | 0.039704 |
| mesencephalon | 0.39664 |
| occipital lobe | -0.36882 |
| parietal lobe | -0.05809 |
| pontine tegmentum | 0.642824 |
| subthalamus | 0.326542 |
| ventral thalamus | 1.037642 |

**Supplementary Table 19:** Quantitation of z scores of gene expression: *PDGFRA*.

**Supplementary Table 20:** Quantitation of z scores of gene expression: *HIST1H3B*.

| **Location** | **Z score** |
| --- | --- |
| basal part of pons | -0.75780278 |
| claustrum | -0.32714258 |
| frontal lobe | -0.32746746 |
| insula | -0.41193055 |
| mesencephalon | 0.13447310 |
| occipital lobe | -0.17003222 |
| parietal lobe | -0.11385503 |
| pontine tegmentum | 0.09494124 |
| temporal lobe | -0.19137821 |
| basal part of pons | -0.75780278 |

**Supplementary Table 21:** Quantitation of z scores of gene expression: *H3F3A*.

| **Location** | **Z score** |
| --- | --- |
| basal part of pons | -1.23554167 |
| claustrum | -0.07348889 |
| frontal lobe | -0.49843645 |
| insula | -0.51951806 |
| mesencephalon | -0.06974152 |
| occipital lobe | -0.35628111 |
| parietal lobe | -0.38285521 |
| pontine tegmentum | -0.18854135 |
| temporal lobe | -0.39032451 |
| basal part of pons | -1.23554167 |

**Supplementary Table 22:** Literature review of common pediatric glioma mutations.

| **First Author and Year** | **Number of patients** | **Mutations found in diffuse midline gliomas** | **Mutations found in hemispheric high-grade gliomas** |
| --- | --- | --- | --- |
| Solomon et al, 2015 | 47 | H3-K27M, IDH1, EGFR, BRAF, p53, ATRX |  |
| Karremann et al, 2018 | 85 | H3-K27M, ATRX, p53 |  |
| Hoffman et al, 2016 | 8 | H3-K27M, ACVR1, PIK3CA, FGFR1, MET, ACVR1, BCOR, ATRX, MYC, TP53 |  |
| Wang et al, 2020 | 43 | H3-K27M, ATRX, p53 |  |
| Fontebasso et al, 2013 | 60 |  | H3.3 G34R/V, IDH1, SETD2 |
| Viaene et al, 2018 | 640 |  | PIK3CA, BRAF, PTEN, SETD2 |
| Mackay et al, 2017 | 1000 | H3.3K27M, BRAF, IDH1 | H3.3 G34R/V, BRAF, IDH1 |
| Salloum et al, 2017 | 16 |  | IDH1, TP53, ACVR1, BRAF, ATRX |
| Garibotto et al, 2020 | 18 | H3.3K27M |  |

**Supplementary Table 23.** Mean Z scores per region. Genes with mutations in midline region.

| **Gene** | **Mean Midline Z score** | **Mean Hemispheric Z score** |
| --- | --- | --- |
| BCORL1 | -0.01380085 | -0.2323215 |
| PPM1D | -0.3153418 | -0.3786554 |
| ASXL1 | -0.1777126 | 0.2117362 |
| ACVR1 | -0.070497 | -0.000225952 |
| PIK3CA | 0.3102653 | -0.2353479 |
| TP53 | 0.08480116 | 0.001031351 |
| Overall | 0.077692153 | -0.105630534 |

**Supplementary Table 24.** Mean Z scores per region. Genes with mutations in hemispheric region.

| **Gene** | **Mean Midline Z score** | **Mean Hemispheric Z score** |
| --- | --- | --- |
| SETD2 | -0.323685 | -0.1655 |
| PTEN | -0.09287154 | 0.2089485 |
| PTPN11 | 0.3525042 | 0.050803 |
| POLE | -0.03822474 | -0.01724291 |
| BLM | -0.2955896 | 0.05512202 |
| ARID1A | 0.02058199 | -0.2571779 |
| APC | -0.01732841 | 0.05630521 |
| ATRX | -0.3084051 | -0.03275029 |
| CDKN2A | 0.07564807 | -0.02878582 |
| MSH6 | 0.1486108 | -0.3317378 |
| PDGFRA | 0.3470656 | 0.04228134 |
| Overall | -0.125746316 | 0.12987575 |

**
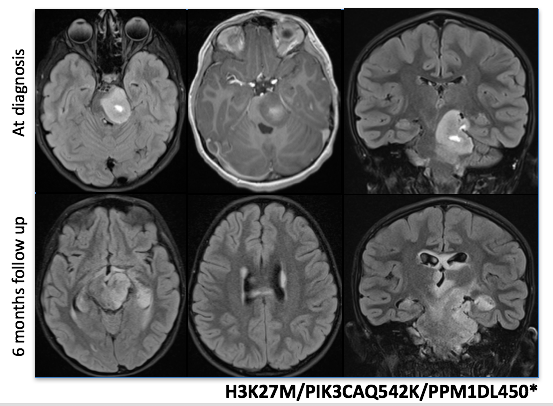
**

**Supplementary Figure 1:** Local and distant metastatic spread by diffuse midline glioma with histone H3 K27M mutation along the midline structures. Additional mutations in PIK3CA and PPM1D were found in the tumor at diagnostic biopsy. Top row left to right: axial FLAIR, axial T1 post-contrast, coronal FLAIR. Bottom row left to right: axial FLAIR, axial FLAIR, coronal FLAIR.


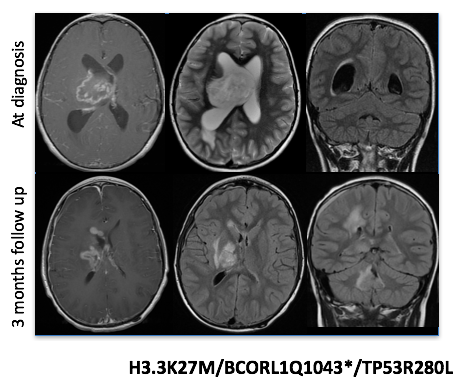


**Supplementary Figure 2:** Local and distant metastatic spread by diffuse midline glioma, H3 K27M-mutant, along the midline structures. Additional mutations in BCORL1 and TP53 were found in the tumor at diagnostic biopsy. Top row left to right: axial T1 post-contrast, axial T2, coronal FLAIR. Bottom row left to right: axial T1 post-contrast, axial FLAIR, coronal FLAIR.

**
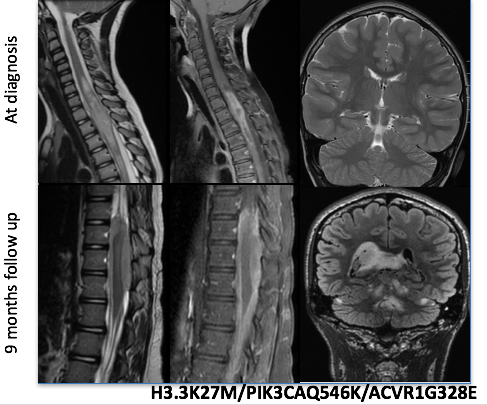
**

**Supplementary Figure 3:** Distant metastatic spread by diffuse midline glioma, H3 K27M-mutant, along the midline structures. Additional mutations in PIK3CA and ACVR1 were found in the tumor at diagnostic biopsy. Top row left to right: sagittal T2 of cervical spine, sagittal T1 post-contrast with fat saturation of cervical spine, coronal T2 of brain. Bottom row left to right: sagittal T2 of cervical spine, sagittal T1 post-contrast of cervical spine, coronal FLAIR of brain.

**
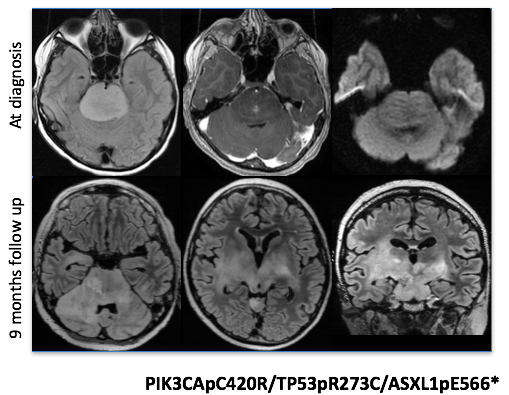
**

**Supplementary Figure 4:** Widespread contiguous infiltrative spread by diffuse midline glioma, H3 K27M-wild- type, for both with H3.3 and H3.1 along the midline structures to the level of cerebral hemisphere. Additional mutations in PIK3CA, TP53, and ASXL1 were found in the tumor at diagnostic biopsy. Top row left to right: axial FLAIR, axial T1 post-contrast, axial DWI. Bottom row left to right: axial FLAIR, axial FLAIR, coronal FLAIR.


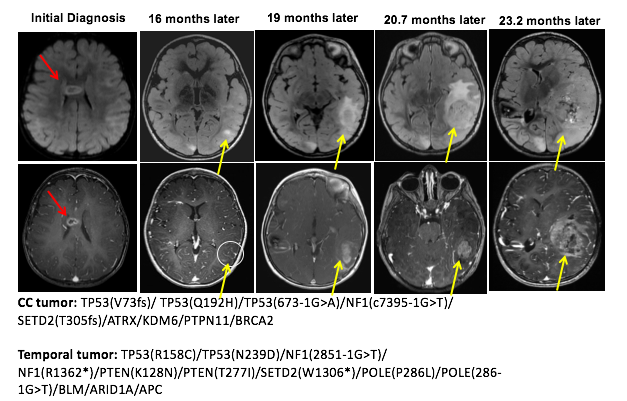


**Supplementary Figure 5:** Multiple separate independently arising hemispheric high-grade glioma in a patient with neurofibromatosis 1. The tumors demonstrated rapid growth and heterogeneous features with enhancement and reduced diffusion. Tumors had multiple mutations with multiple types of mutations in TP53, NF1, PTEN, and SETD2. Top row: axial FLAIR. Bottom row: axial T1 post-contrast.

The second patient with hemispheric high-grade glioma demonstrated inactivating mutations in TP53 and PIK3CA. These mutations were similar to what is typically seen in diffuse midline gliomas but lacked a mutation in a chromatin modification related pathway. This patient was diagnosed at 2.8 years of age and was found to have a left temporal FLAIR hyperintense expansile mass that showed minimal initial enhancement and heterogeneous diffusion characteristics with regions of low and high ADC values (Supplementary Figure 6). This tumor did not undergo gross total resection and demonstrated significant local progression within 6 months of diagnosis. On six months follow up, the tumor demonstrated heterogeneous enhancement and local spread into the adjacent basal ganglia, corpus callosum, and cerebral peduncles. The patient lived for 7 months after diagnostic biopsy.


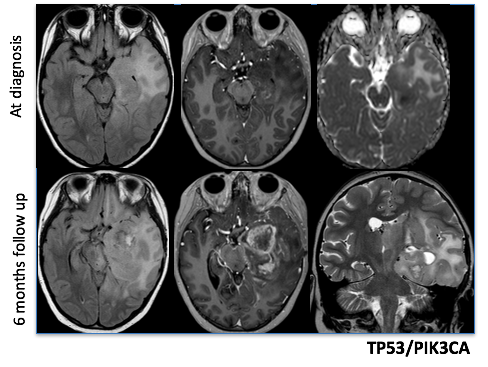


**Supplementary Figure 6:** Hemispheric high-grade glioma with inactivating mutations in TP53 and PIK3CA. The tumor demonstrates aggressive infiltrative spread and invasion into adjacent basal ganglia and subinsular white matter. Top row left to right: axial FLAIR, axial T1 post-contrast, axial ADC. Bottom row left to right: axial FLAIR, axial T1 post-contrast, coronal T2.


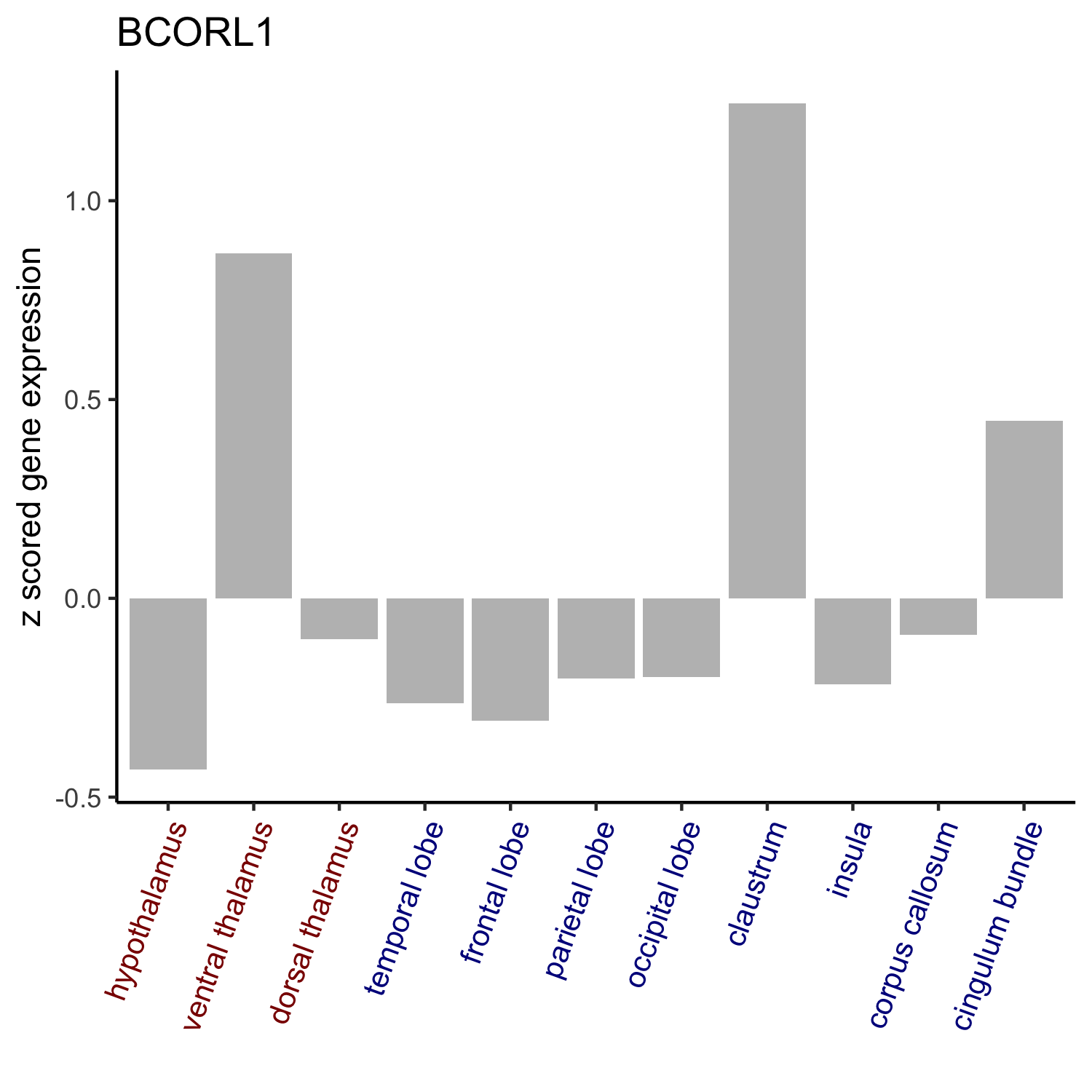


**Supplementary Figure 7A:** Quantitation of z scores of gene expression: BCORL1.


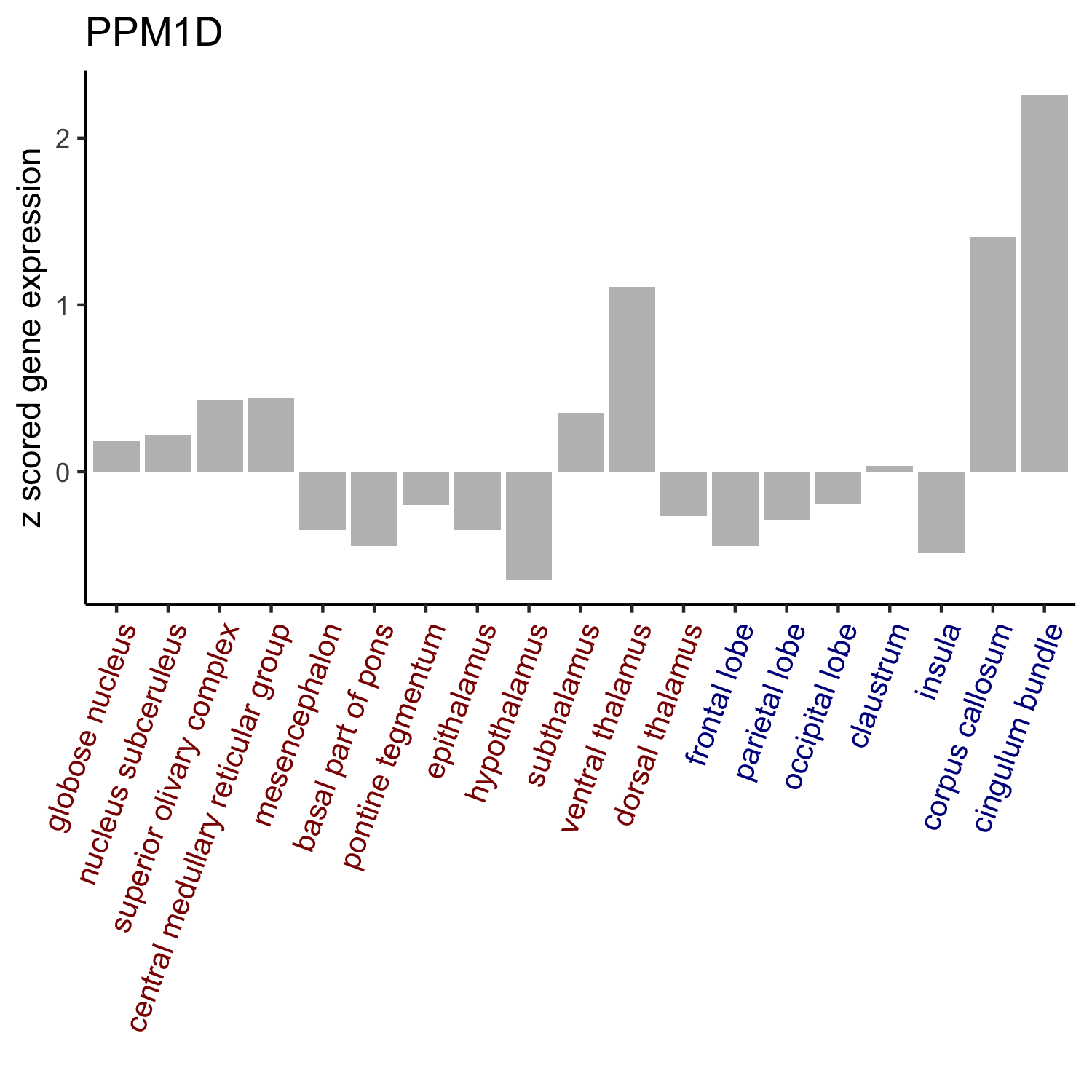


**Supplementary Figure 7B:** Quantitation of z scores of gene expression: PPM1D.


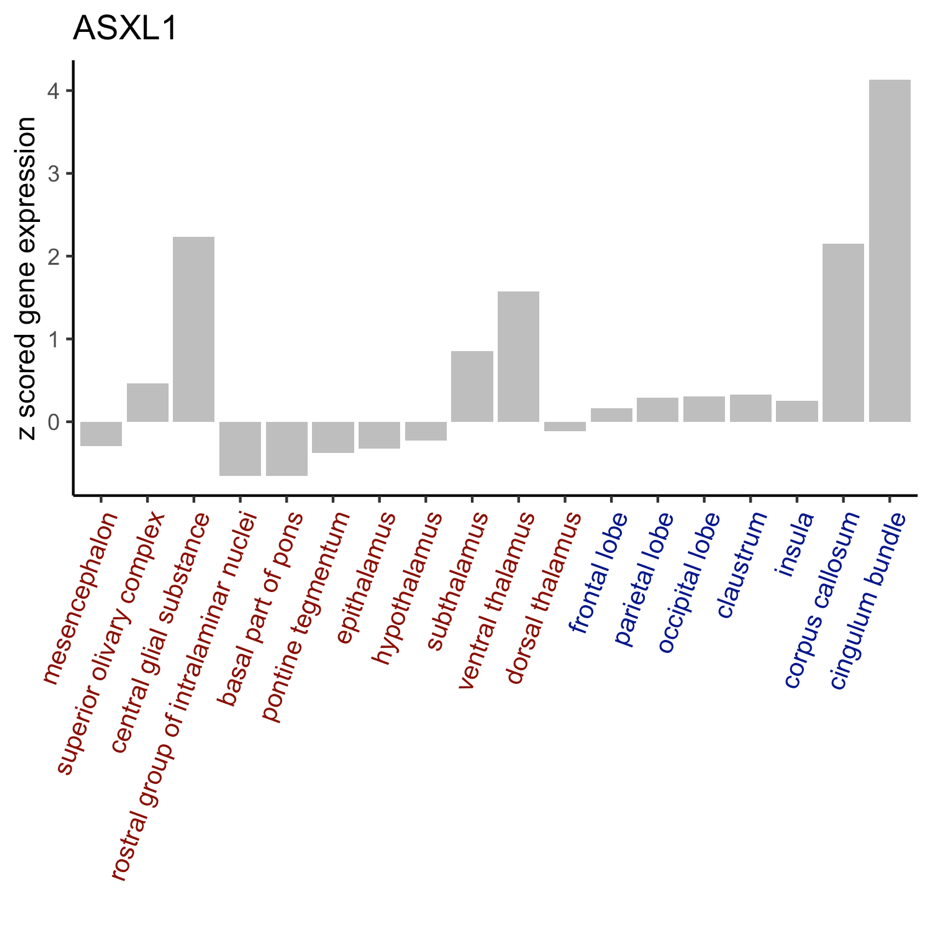


**Supplementary Figure 7C:** Quantitation of z scores of gene expression: ASXL1.


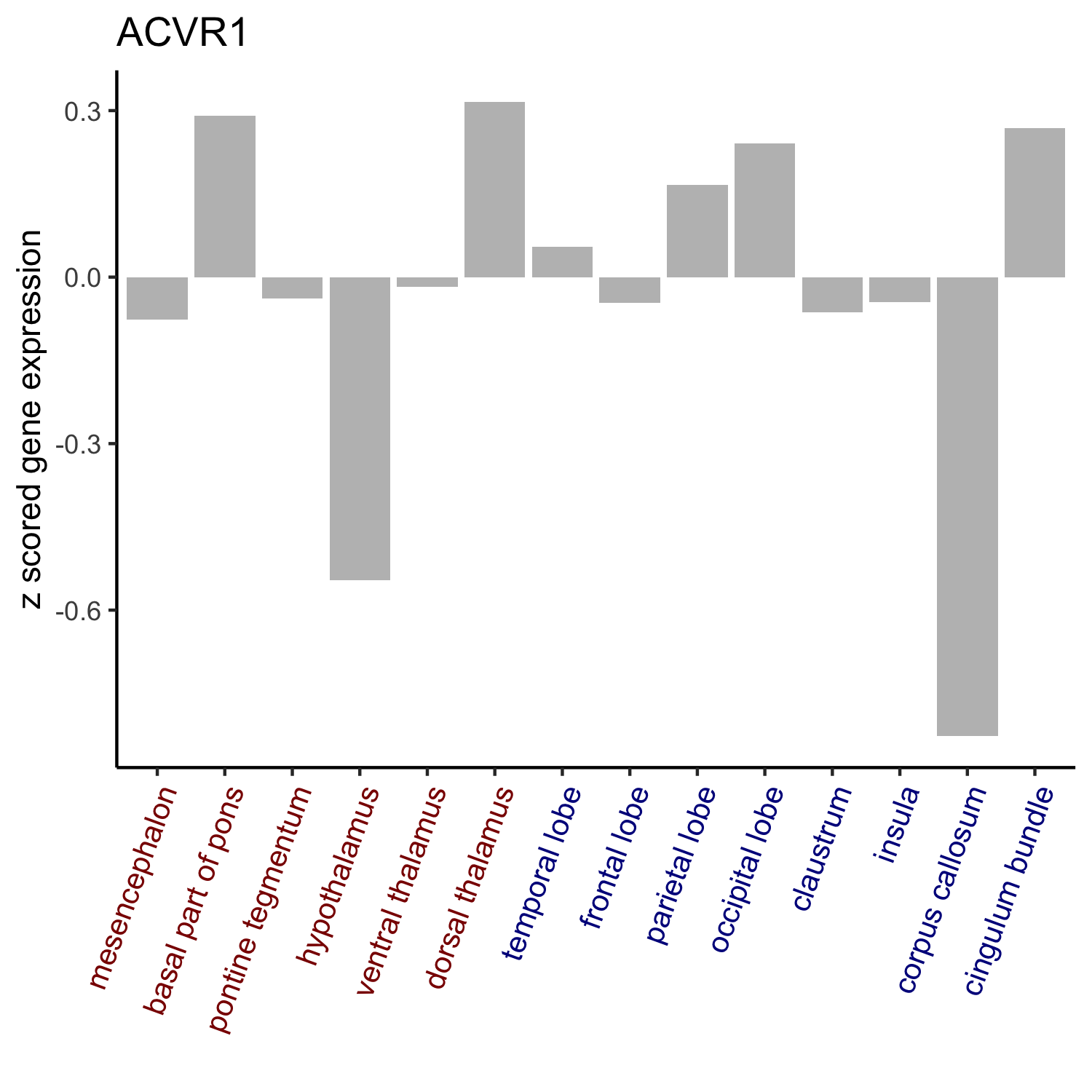


**Supplementary Figure 7D:** Quantitation of z scores of gene expression: ACVR1.


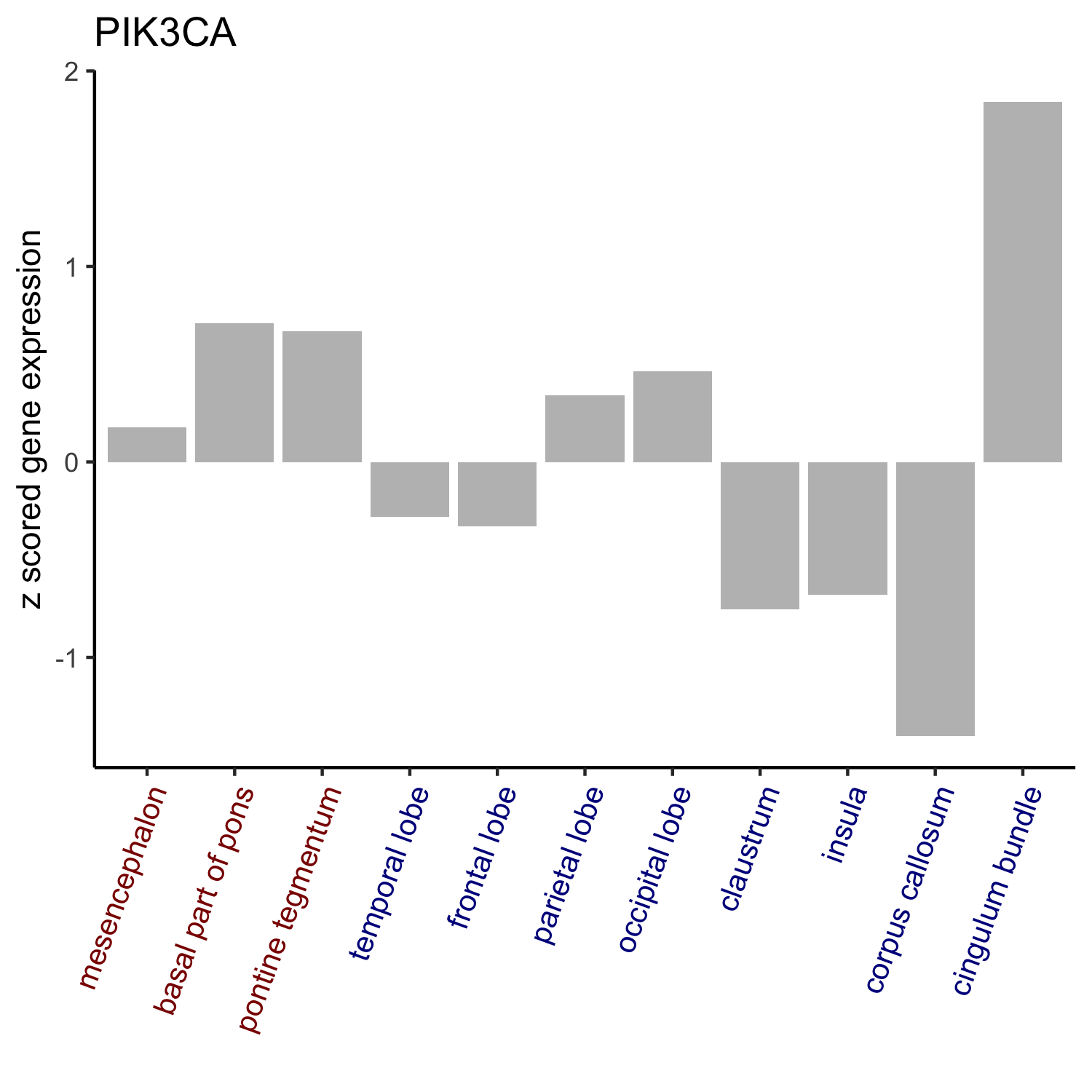


**Supplementary Figure 7E:** Quantitation of z scores of gene expression: PIK3CA.


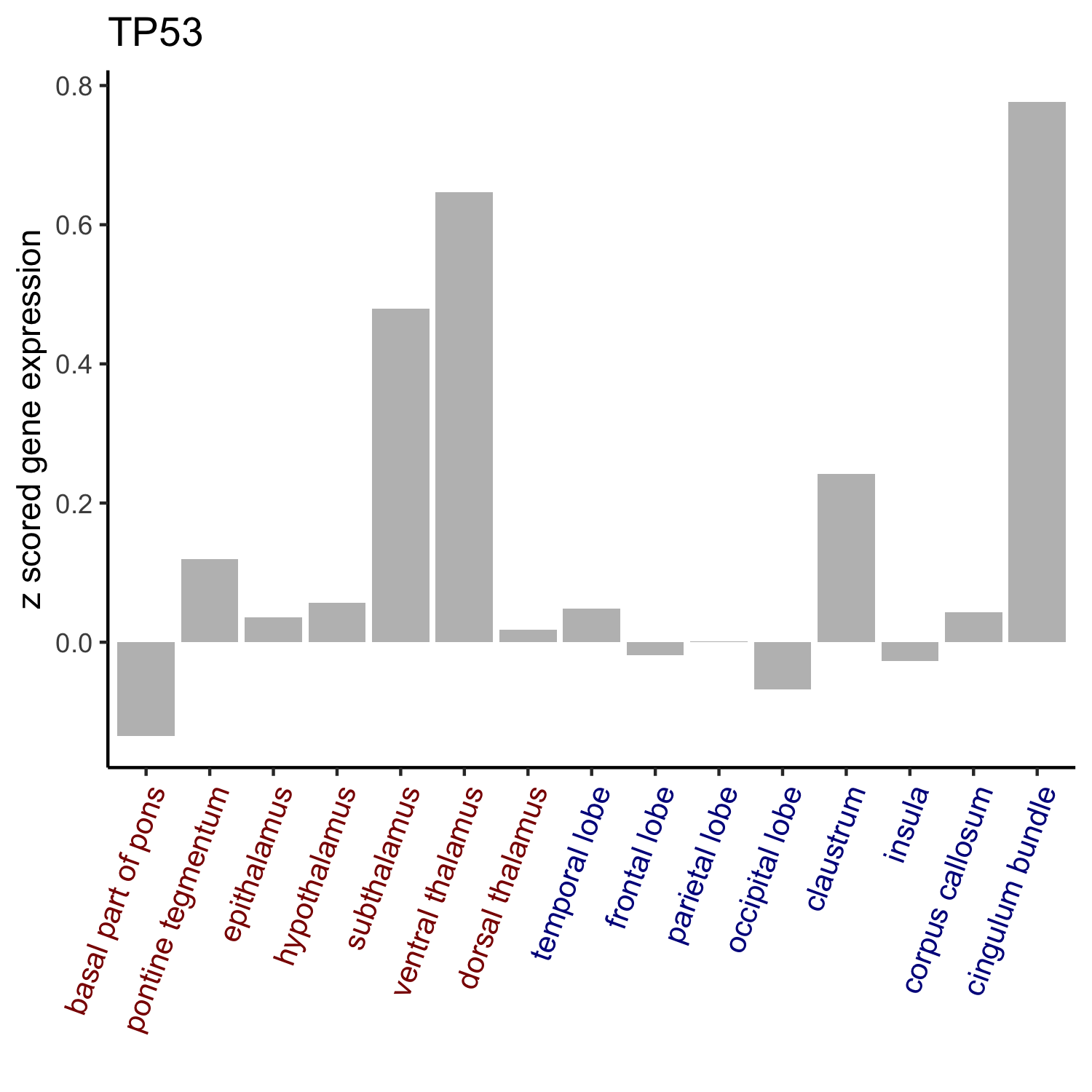


**Supplementary Figure 7F:** Quantitation of z scores of gene expression: TP53.


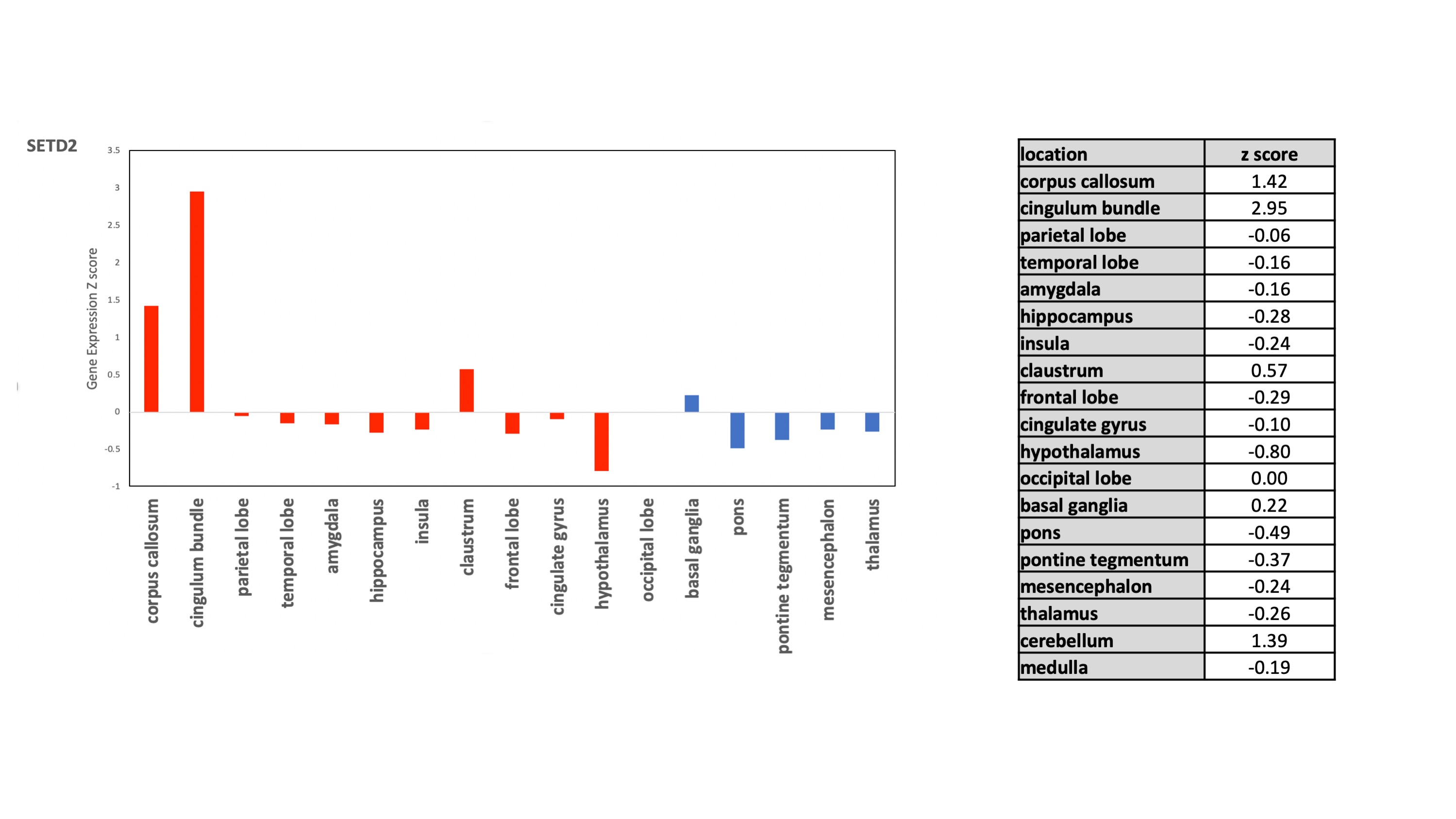


**Supplementary Figure 8A:** Quantitation of z scores of gene expression: SETD2.

_
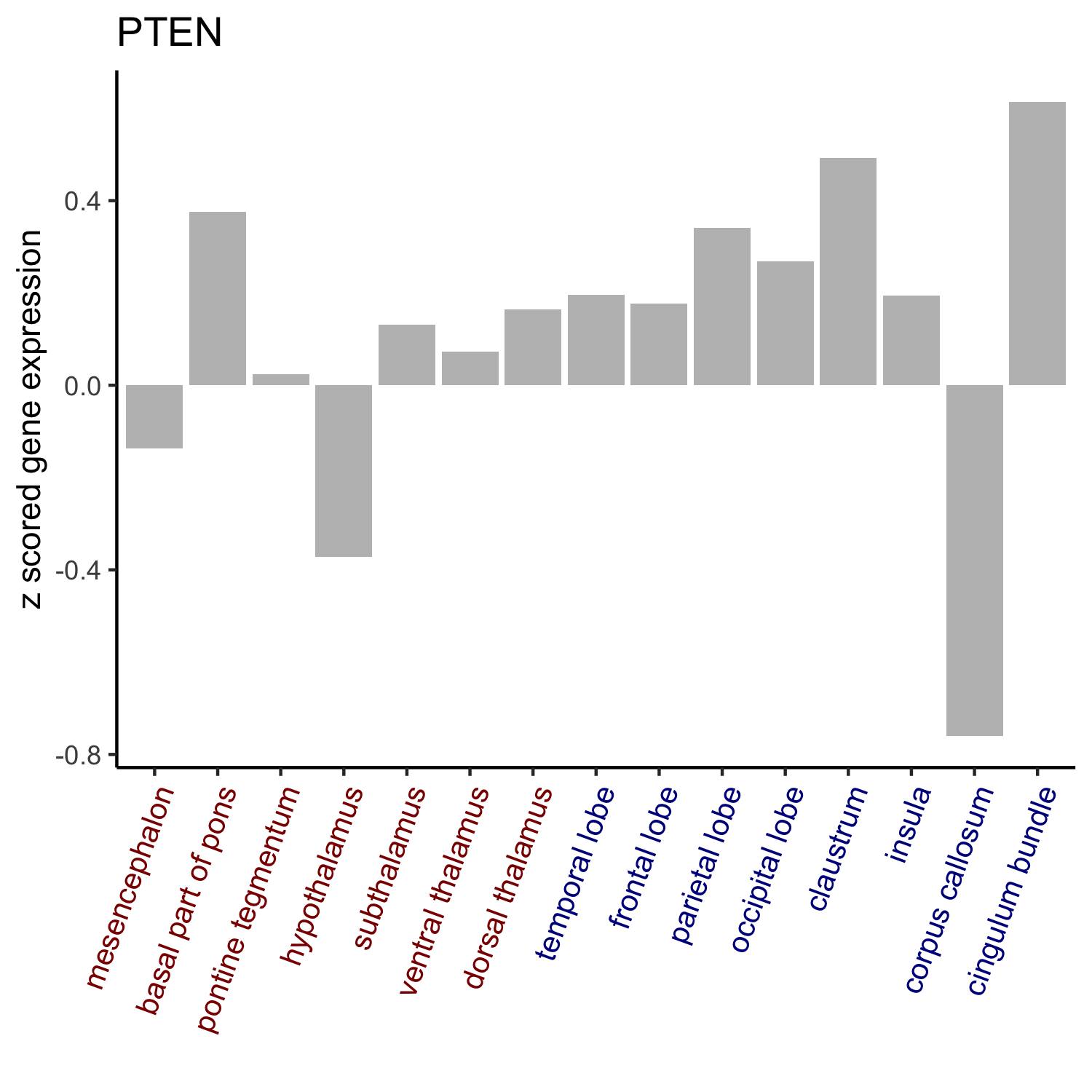
_

**Supplementary Figure 8B:** Quantitation of z scores of gene expression: PTEN.


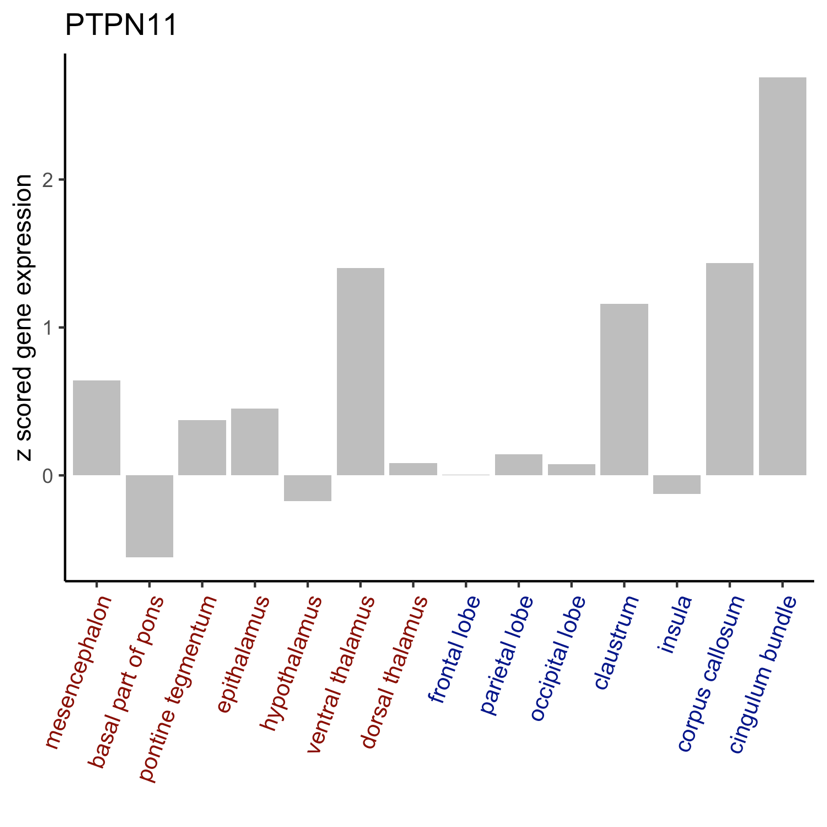


**Supplementary Figure 8C:** Quantitation of z scores of gene expression: PTPN11.


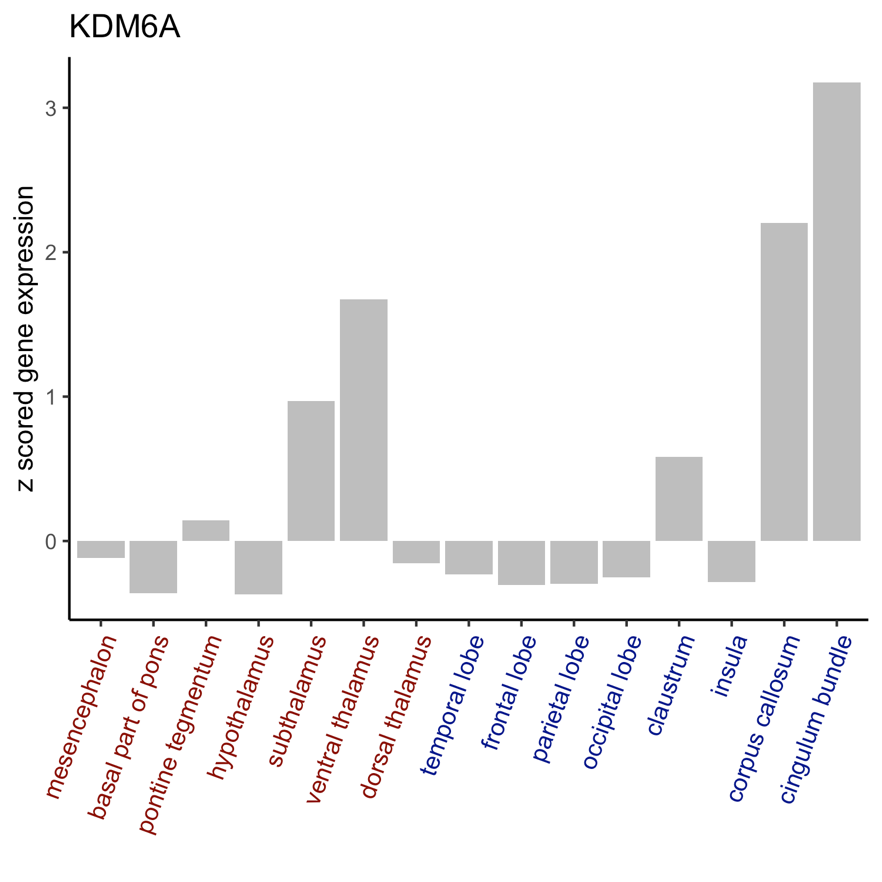


**Supplementary Figure 8D:** Quantitation of z scores of gene expression: KDM6A.


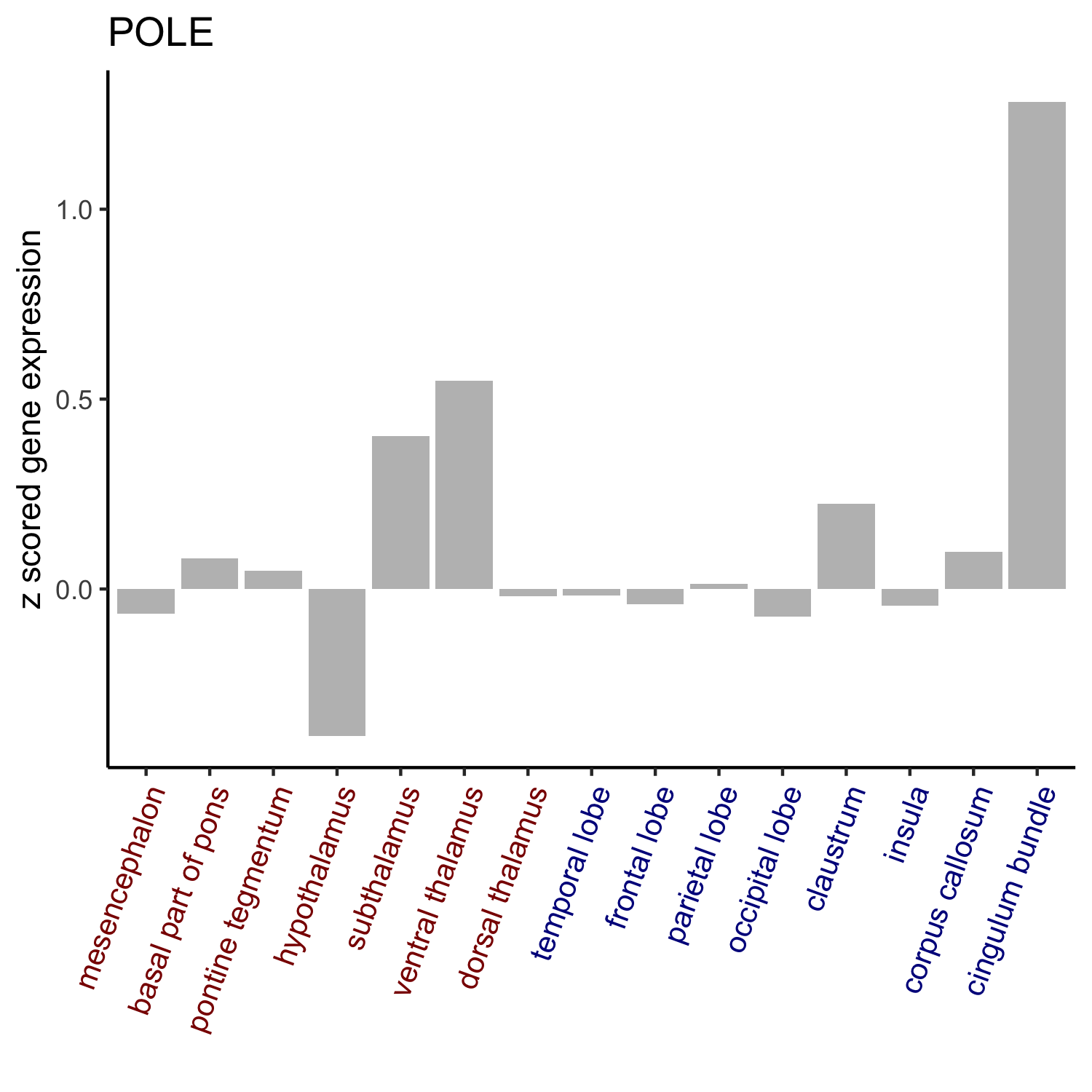


**Supplementary Figure 8E:** Quantitation of z scores of gene expression: POLE.


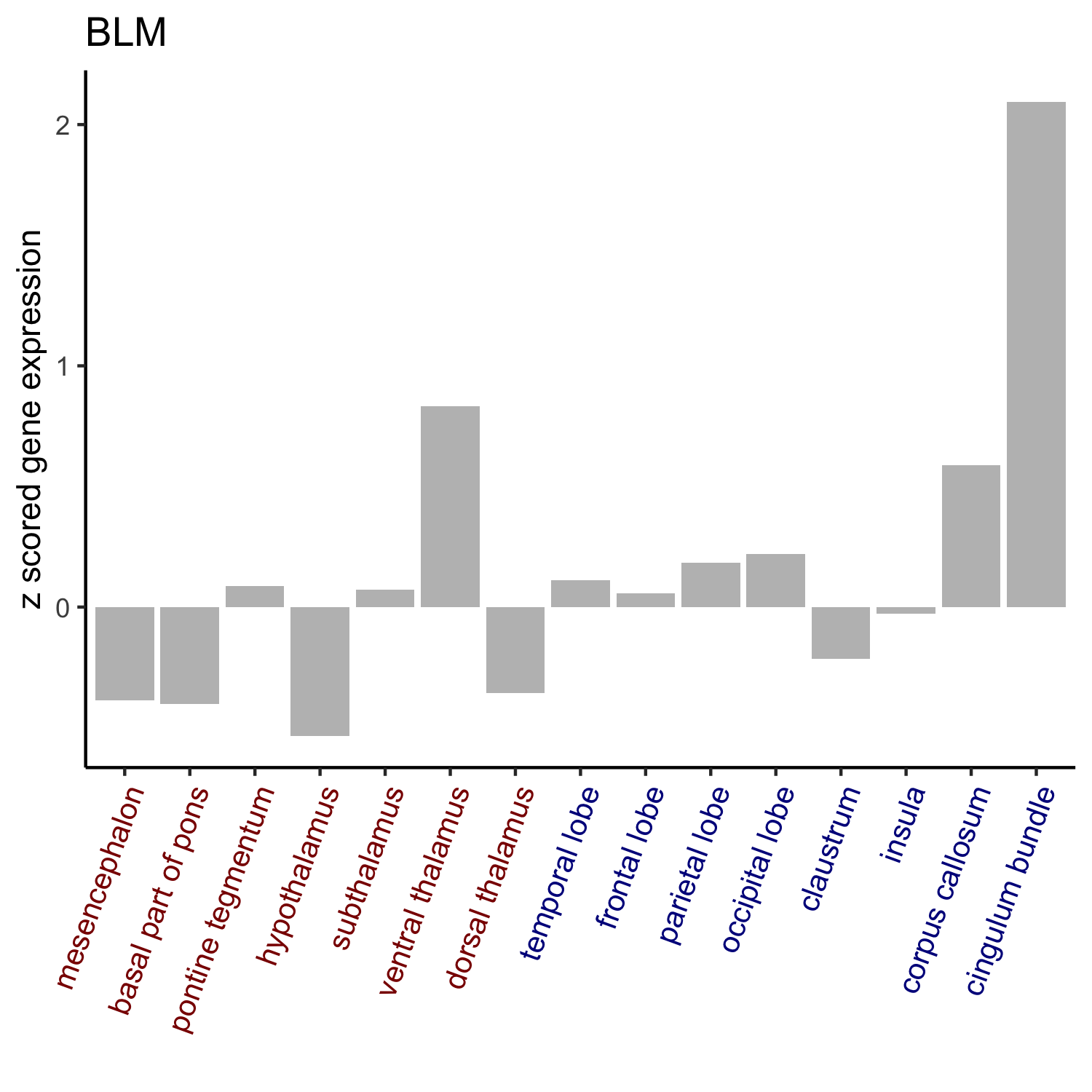


**Supplementary Figure 8F:** Quantitation of z scores of gene expression: BLM.


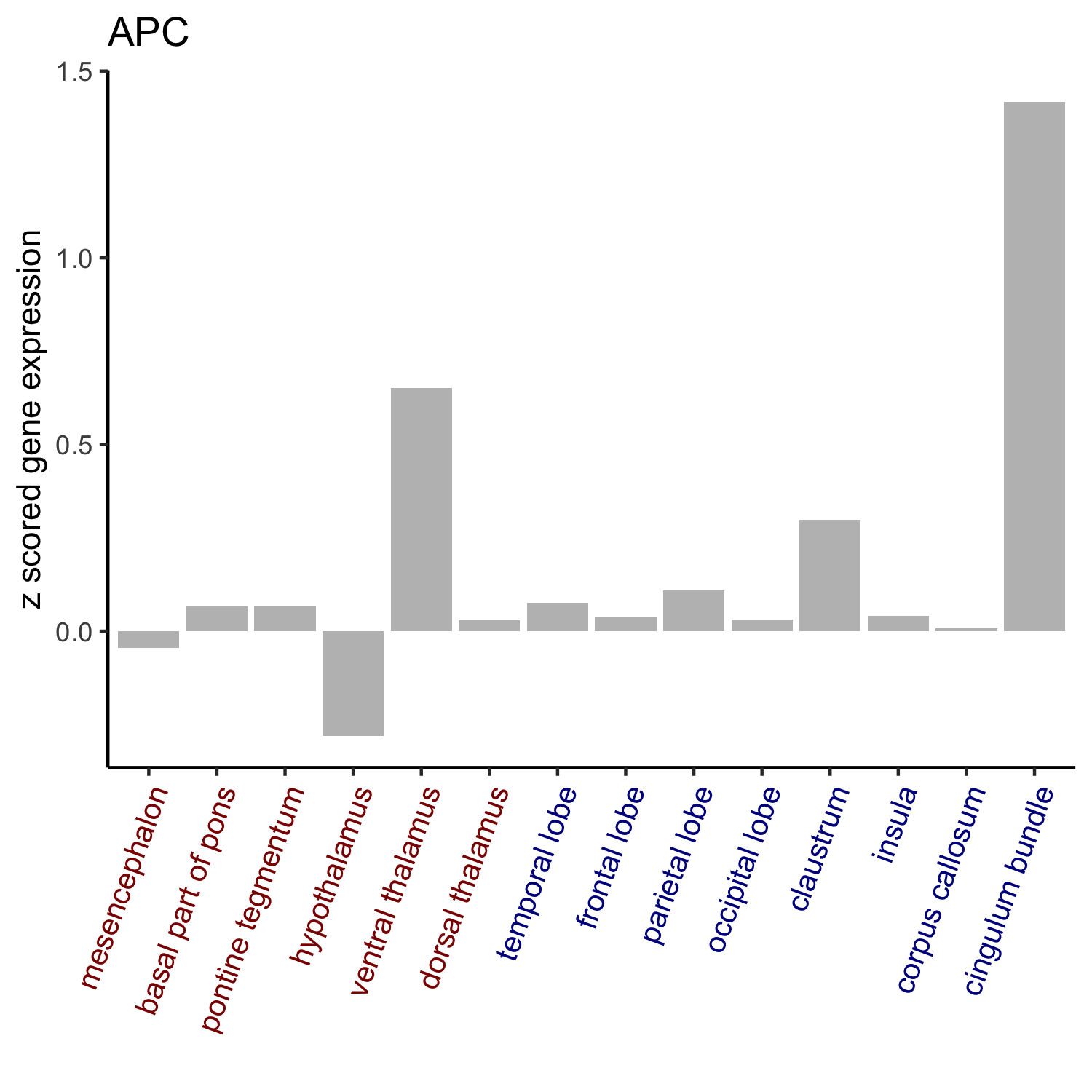


**Supplementary Figure 8G:** Quantitation of z scores of gene expression: ARID1A.


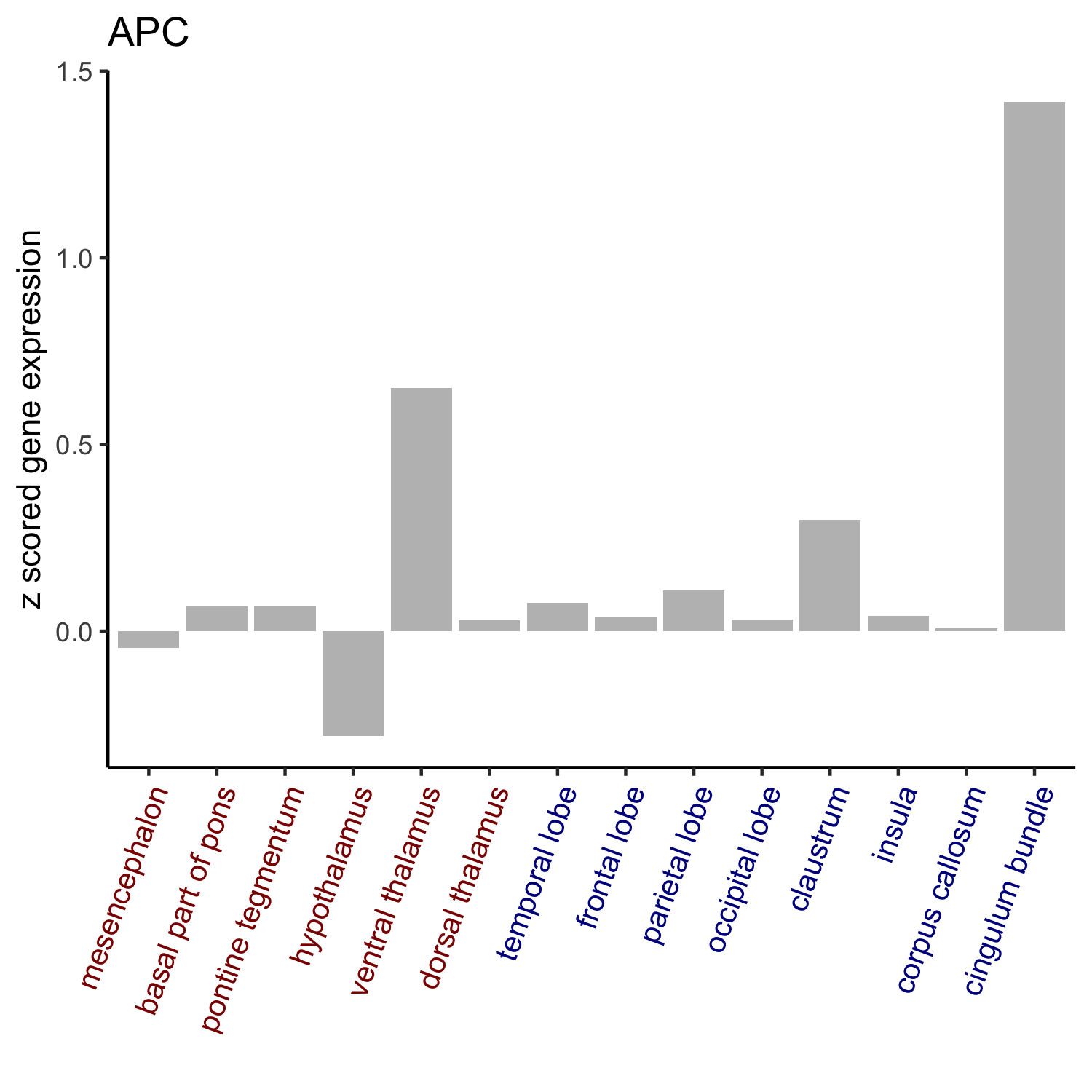


**Supplementary Figure 8H:** Quantitation of z scores of gene expression: APC.


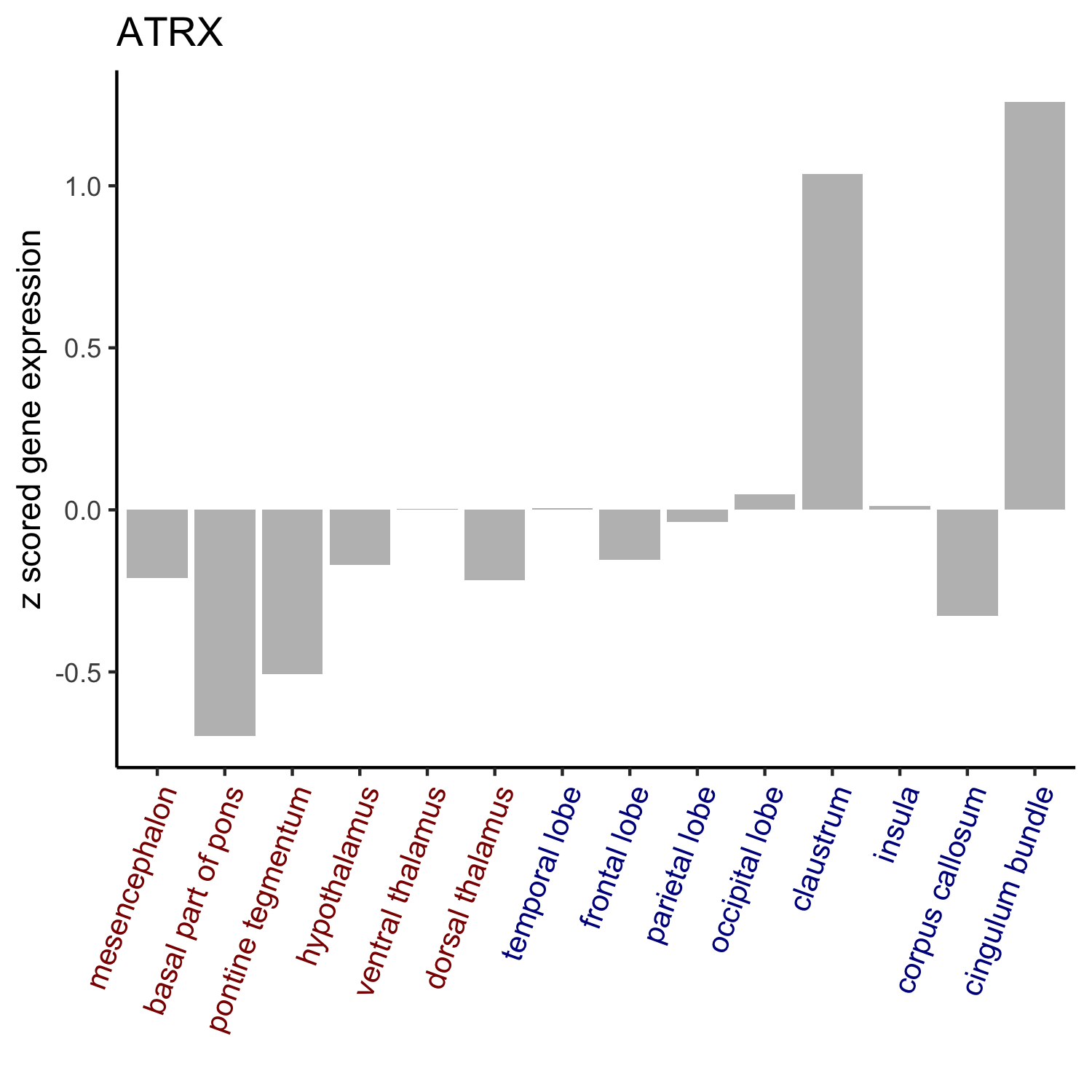


**Supplementary Figure 8I:** Quantitation of z scores of gene expression: ATRX.


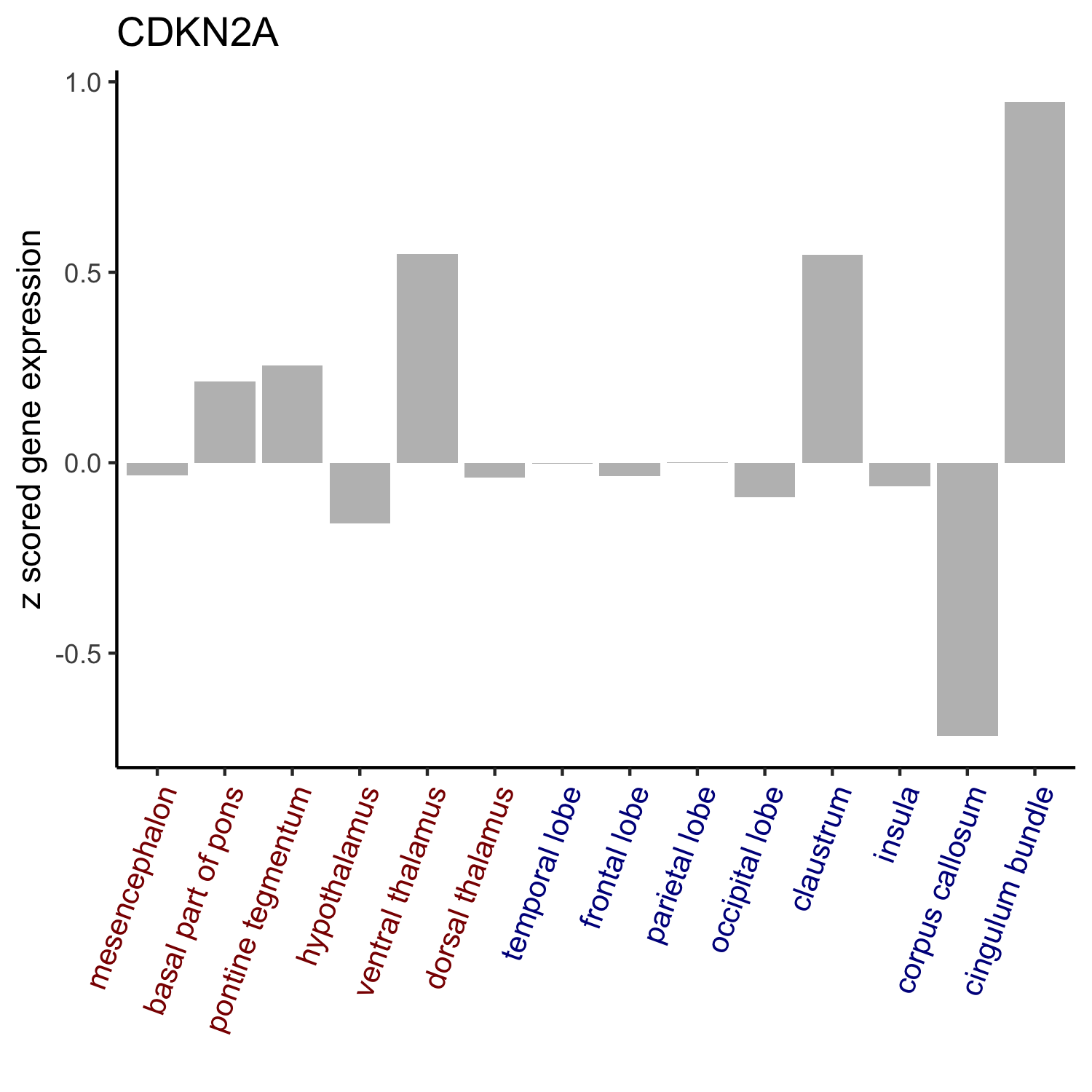


**Supplementary Figure 8J:** Quantitation of z scores of gene expression: CDKN2A.


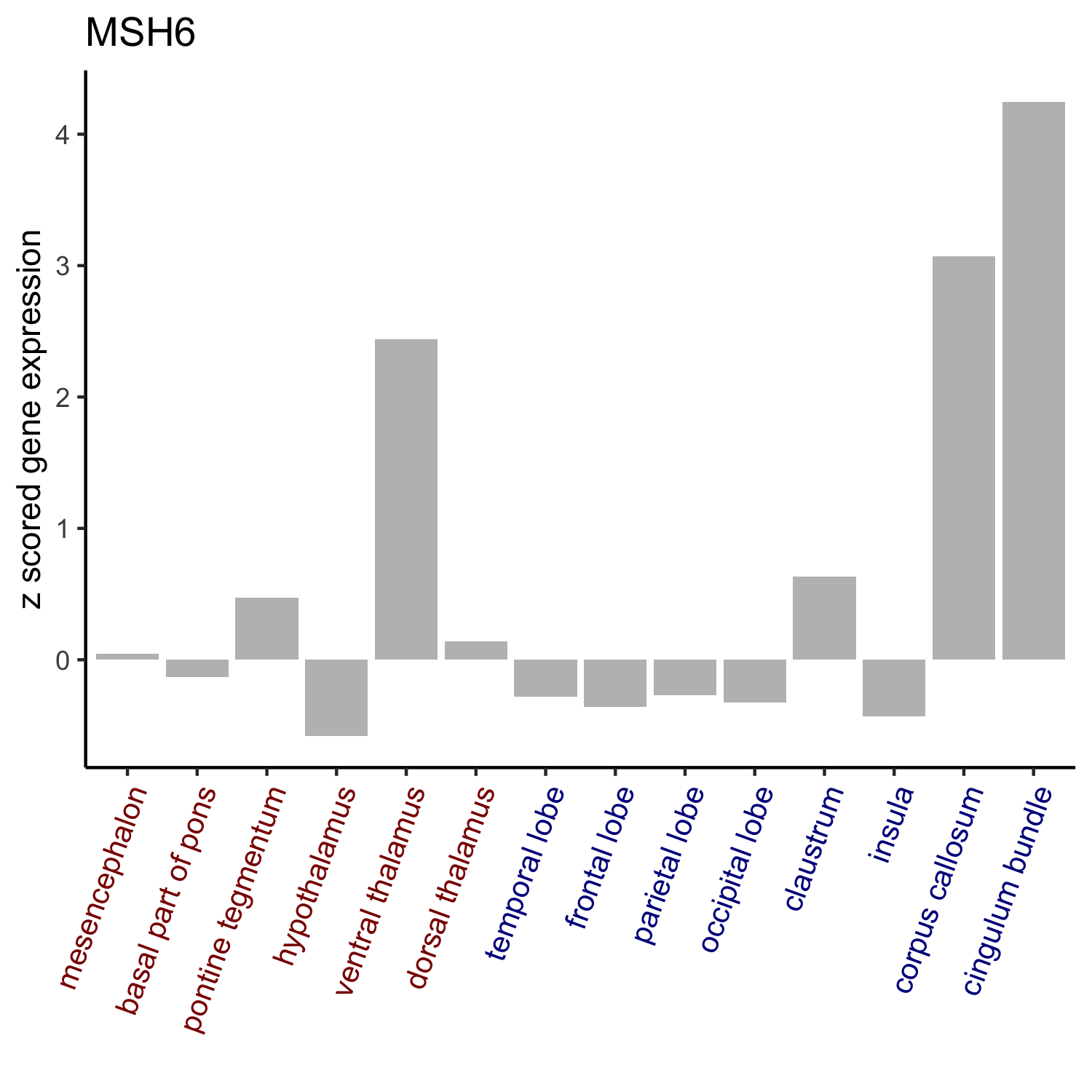


**Supplementary Figure 8K:** Quantitation of z scores of gene expression: MSH6.

**
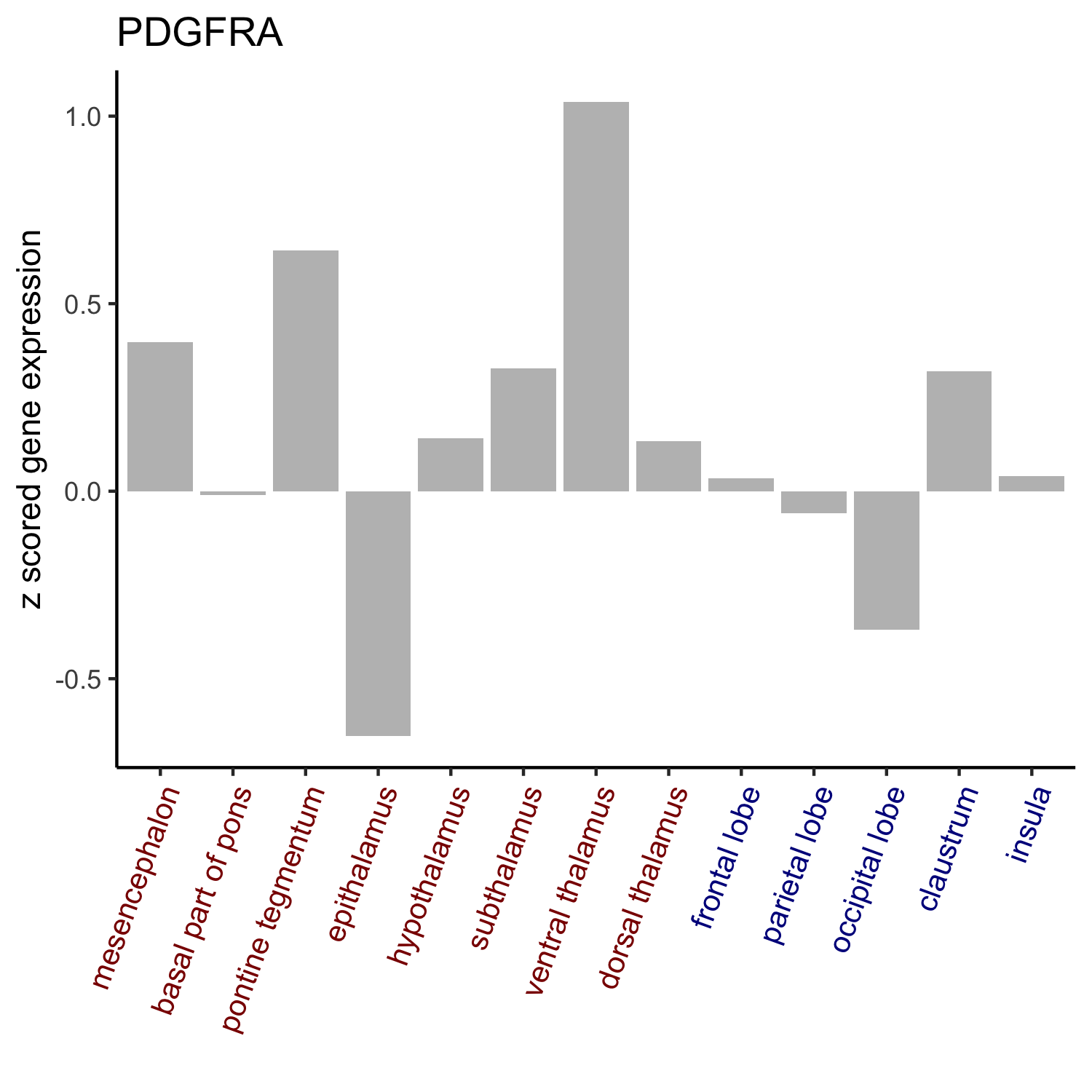
**

**Supplementary Figure 8L:** Quantitation of z scores of gene expression: PDGFRA.


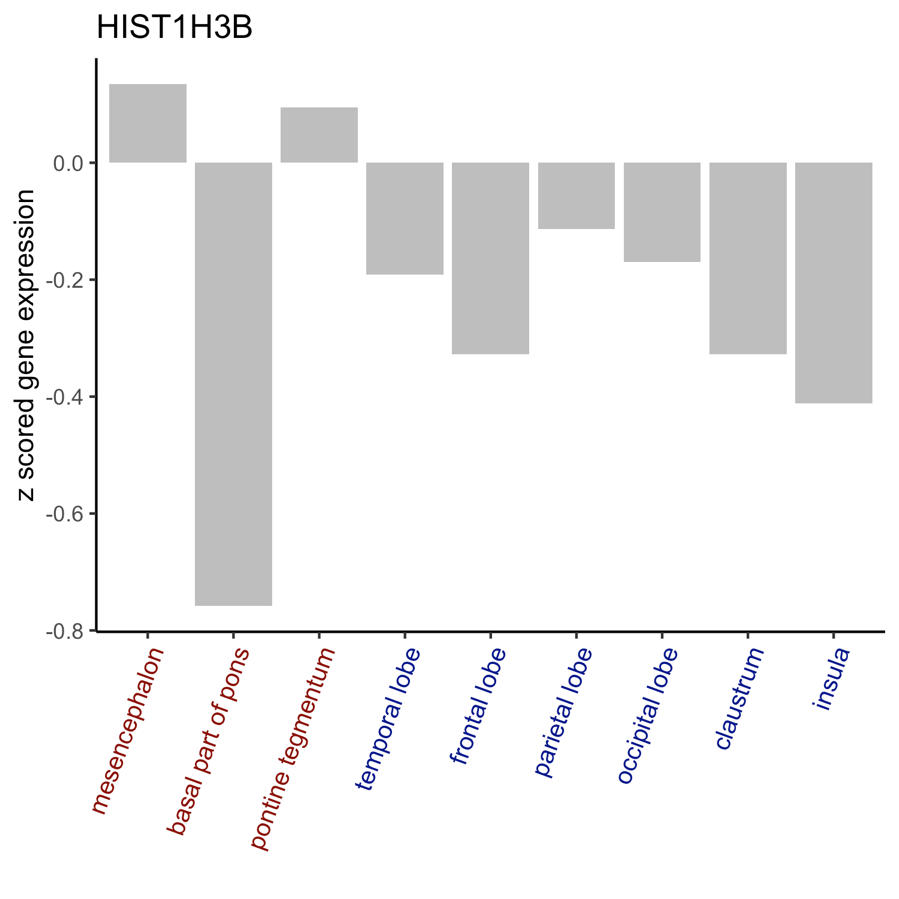


**Supplementary Figure 8M:** Quantitation of z scores of gene expression: HIST1H3B.


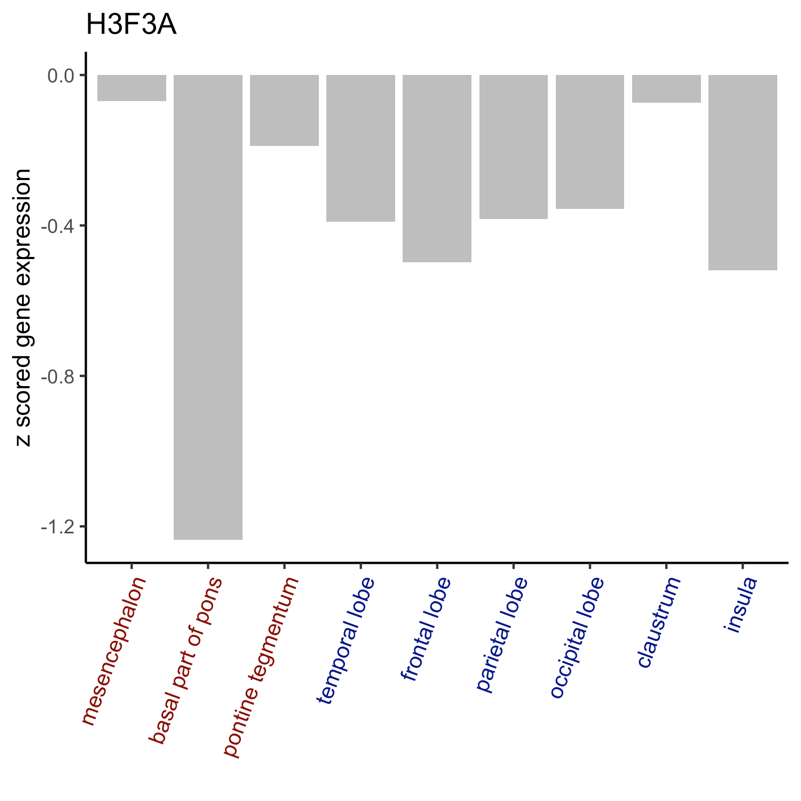


**Supplementary Figure 8N:** Quantitation of z scores of gene expression: H3F3A.


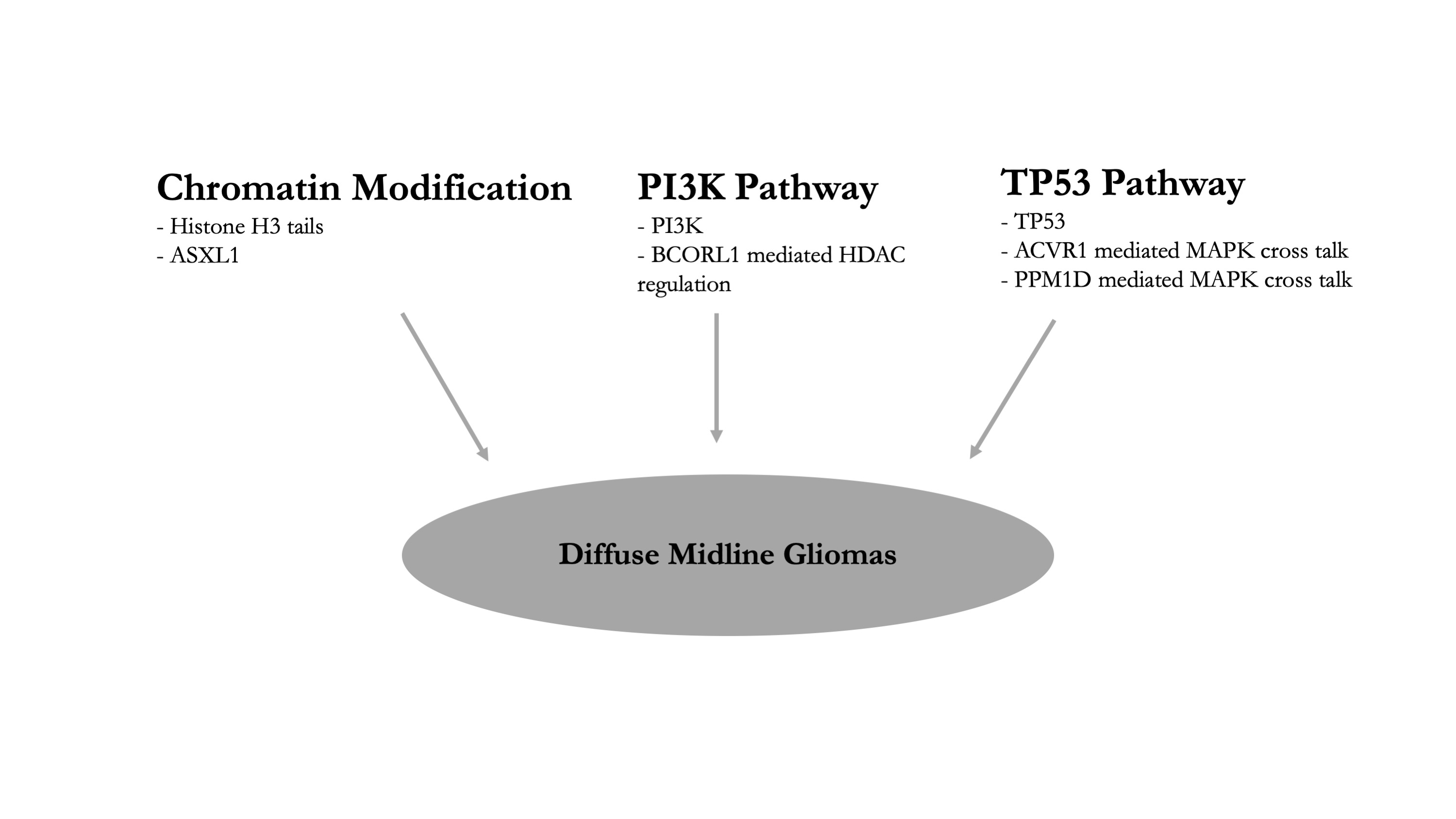


**Supplementary Figure 9:** Genetic mutations within diffuse midline gliomas fall within 3 distinct pathways that act in coordination with each other. Tumors were always found with alteration in chromatin modification and gene expression and an additional mutation in the PI3K pathway and the TP53 pathway.
